# Advances and challenges in the use of liquid biopsy in gynaecological oncology

**DOI:** 10.1016/j.heliyon.2024.e39148

**Published:** 2024-10-15

**Authors:** Yingfeng Zhang, Libi Tian

**Affiliations:** University-Town Hospital of Chongqing Medical University, Chongqing, 401331, China

**Keywords:** Gynaecological cancers, Liquid biopsy, ctDNA, CTCs, cfRNA, TEPs, Exosomes

## Abstract

Ovarian cancer, endometrial cancer, and cervical cancer are the three primary gynaecological cancers that pose a significant threat to women's health on a global scale. Enhancing global cancer survival rates necessitates advancements in illness detection and monitoring, with the goal of improving early diagnosis and prognostication of disease recurrence. Conventional methods for identifying and tracking malignancies rely primarily on imaging techniques and, when possible, protein biomarkers found in blood, many of which lack specificity. The process of collecting tumour samples necessitates intrusive treatments that are not suitable for specific purposes, such as screening, predicting, or evaluating the effectiveness of treatment, monitoring the presence of remaining illness, and promptly detecting relapse. Advancements in treatment are being made by the detection of genetic abnormalities in tumours, both inherited and acquired. Newly designed therapeutic approaches can specifically address some of these abnormalities. Liquid biopsy is an innovative technique for collecting samples that examine specific cancer components that are discharged into the bloodstream, such as circulating tumour DNA (ctDNA), circulating tumour cells (CTCs), cell-free RNA (cfRNA), tumour-educated platelets (TEPs), and exosomes. Mounting data indicates that liquid biopsy has the potential to improve the clinical management of gynaecological cancers through enhanced early diagnosis, prognosis prediction, recurrence detection, and therapy response monitoring. Understanding the distinct genetic composition of tumours can also inform therapy choices and the identification of suitable targeted treatments. The main benefits of liquid biopsy are its non-invasive characteristics and practicality, enabling the collection of several samples and the continuous monitoring of tumour changes over time. This review aims to provide an overview of the data supporting the therapeutic usefulness of each component of liquid biopsy. Additionally, it will assess the benefits and existing constraints associated with the use of liquid biopsy in the management of gynaecological malignancies. In addition, we emphasise future prospects in light of the existing difficulties and investigate areas where further research is necessary to clarify its rising clinical capabilities.

## 1 Liquid biopsy

The prevailing trajectory in the field of oncology is shifting towards the use of minimally invasive methodologies for the timely identification, continuous surveillance, and prognostication of therapeutic outcomes in individuals afflicted with cancer [1]. The present approaches employed for the analysis and surveillance of gynaecological malignancies predominantly depend on conventional biopsy, an invasive technique that plays a crucial role in the diagnostic and prognostic evaluation process. While conventional biopsy has long been regarded as the preferred method for disease surveillance [2], its intrusive nature and limited ability to capture tumour heterogeneity render it an inadequate tool [3]. The aforementioned fact provides a rationale for the scientific community's endeavour to identify novel non-invasive techniques for longitudinal sampling [4]. Due to recent efforts, liquid biopsy analysis has become a valuable non-invasive method for comprehensively and dynamically studying the molecular features of tumours [2]. In comparison to conventional biopsy techniques, liquid biopsy offers the advantage of providing real-time data on the tumour, enabling the continuous monitoring of its progression and its reaction to therapeutic interventions [2].

The field of precision oncology has arisen in order to get more precise and efficacious therapies for specific patients by leveraging the molecular attributes of the tumour. The field of personalised oncology is closely linked to the identification of molecular biomarkers that are valuable in forecasting tumour prognosis and medication efficacy, as well as achieving precise disease surveillance. Within this particular framework, the use of circulating biomarkers and liquid biopsies plays a crucial role in the implementation of personalised oncology, serving as an optimal adjunct to tissue biopsies and radiologic examinations. Utilising circulating biomarkers significantly enhances the evaluation of tumour irregularities and changes in both space and time [5].

**Fig. 1:**
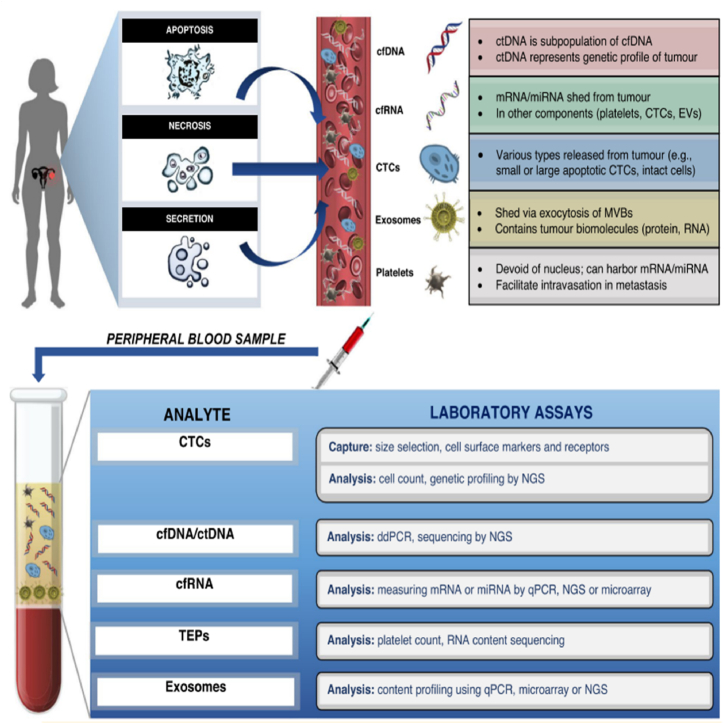
Overview of the liquid biopsy process, from hypothesized mechanisms of tumour release of liquid biopsy components, to laboratory analysis techniques. Tumour biomarkers are first released and enter the circulation via one of three main mechanisms: apoptosis, necrosis, or secretion. Liquid biopsy involves the collection and analysis of five distinctive tumour components from peripheral blood samples: cell-free nucleic acids (cfDNA/ctDNA, cfRNA), CTCs, exosomes and tumour educated platelets. Tumour components in peripheral blood samples are then captured and analysed using their corresponding laboratory assays [8].

A liquid biopsy, alternatively referred to as fluid biopsy or fluid phase biopsy, involves the collection and examination of non-solid biological tissue to identify tumour-related components, including circulating tumour cells, ctDNA, EVs, cell-free microRNAs (cfmiRNAs), mRNA, long noncoding RNAs (lncRNAs), small RNA, circulating cell-free proteins, and TEPs [6] (Table 1). Various bodily fluids, such as blood, urine, uterine aspirates, saliva, cerebrospinal fluid, and pleural effusions, can be used for liquid biopsy. The optimal treatment for a patient's tumour depends on its molecular profile. However, the neoplastic molecular profile can change as the malignancy evolves, and substantial variability can exist even within a single tumor. A needle biopsy may not be suitable for accessing all tumours and their metastases, making it challenging to treat a tumour based on its distinct molecular signature at any given point. One solution is the use of liquid biopsy, which provides convenient access to tumour cells or tumoural products at any time. Serial liquid biopsies offer a more effective method for monitoring the geographical and temporal heterogeneity of tumours. This enhanced capability enables more accurate tracking of the disease's progression and presents opportunities for the advancement of more effective treatment options (Fig. 1) [7].

**Table 1:** Comparison of various types of liquid biopsies.

Marker	Definition	Strengths	Weaknesses	Applications
CTCs	Tumour cells detached from solid tumours and entering the peripheral circulation	1.Specific for tumour origin 2.DNA, RNA and proteins are available 3. 3.Capable of in vitro testing	1.Specificity of CTC identification 2.Impact of CTC heterogeneity on enrichment methods 3.Implications of CTCs for medication guidance	1.Prognostic analysis 2.Efficacy assessment 3.Recurrence monitoring
CtDNA/cfRNA	Circulating DNA/RNA released from tumour cells into the bloodstream	1.comprehensively reflecting the information of tumour mutation 2.high specificity of tumour-associated mutations and clear relationship with targeted drugs	1.Difficult to identify tissue origin 2.Mutation frequency is usually low, and it is technically difficult to distinguish between tumour variants and background noise.	1.Personalised medication guidance 2.Efficacy and resistance monitoring 3.Early companion diagnostics
Exosomes	Tumour cells shed vesicles that carry information about the DNA, RNA and proteins of the cancer cells	1.Non-coding RNA, DNA and proteins are available 2. Capable of in vitro testing 3.Broad applicability and greater potential as early screening markers	1. There is still a lack of clear specific markers. 2.Further purification of tumour exosomes is challenging	1.Early screening 2.Metastasis regulation

Liquid biopsy has been widely used in oncology research for over 20 years due to the advancement of sensitive techniques for identifying tumour material. However, the main constraint is the limited amount of tumour material in circulation. In patients with metastasis, the average level of CTC is 1 CTC/10^6−8^, with a concentration of ctDNA often below 0.01 %. Today, sensitive procedures can address liquid biopsy analyses with sufficient assurance [9]. CTC research is considered the foundational stage in liquid biopsy, as cells are released into the bloodstream during primary tumour development. CTCs have been proven as a prognostic indicator in metastatic breast cancer and other solid tumours, showing superior accuracy compared to traditional imaging techniques. However, CTC monitoring for early detection of minimal residual illness still faces technological challenges.

However, the molecular characterization of CTCs holds significant importance in informing the choice of targeted therapies. This is because it enables doctors to gain a comprehensive understanding of several molecular targets, including but not limited to ERBB2, EGFR, AR, and PD-L1 [10].

Although numerous studies have shown the significance of CTCs in cancer treatment, the outcomes of clinical trials have not provided a definitive therapeutic advantage. By contrast, the ctDNA, which is the younger counterpart of CTCs, has been successfully integrated into regular clinical procedures following the approval of the EGFR mutation test (Therascreen EGFR Plasma, Qiagen) by the European Medicines Agency (EMA) in the plasma of patients diagnosed with non-small cell lung cancer (NSCLC) [11]. Experts have devised various techniques to identify ctDNA with high sensitivity and specificity. These techniques encompass beads, emulsion, amplification, and magnetics-based digital PCR (BEAMing) [12], safe sequencing (Safe-Seq) [13], tagged amplicon deep sequencing (TAm-Seq) [14], and digital PCR [15]. These methods are employed for the detection of point mutations or whole-genome sequencing [16]. Several studies have utilised these technologies to illustrate the potential utility of ctDNA as a valuable tool in drug development and the examination of intratumor heterogeneity and clonal evolution in several types of cancers, including breast, colon, melanoma, and NSCLC [11]. TAm-Seq has shown a high level of sensitivity in detecting point mutations, particularly in gynaecological malignancies such as ovarian cancer [17].

Conversely, there is a growing interest in the characterization of circulating exosomes and miRNAs. Tumour entities have a role in the development and spread of cancer, and detecting them in different body fluids is a promising approach to uncovering specific biomarkers that are relevant for diagnosis and prognosis. One further benefit of circulating exosomes in comparison to CTCs or ctDNA is their ability to offer larger quantities of tumour material for genetic analysis. System Bioscience has developed numerous commercial kits, such as ExoQuick, to enhance and streamline the isolation process. In addition, it has been reported that tumour exosome biomarkers, including HSP60 and GPC1, hold significant potential as potential candidates for the diagnosis of colorectal, pancreatic, and breast cancer. Nevertheless, the investigations on exosomes and miRNA in blood are now in an exploratory stage. It is imperative to conduct additional clinical research with standardised protocols to validate these biomarkers before they can be routinely utilised in clinical settings [18].

### 1.1 Circulating tumor cells (CTCs)

**Fig. 2:**
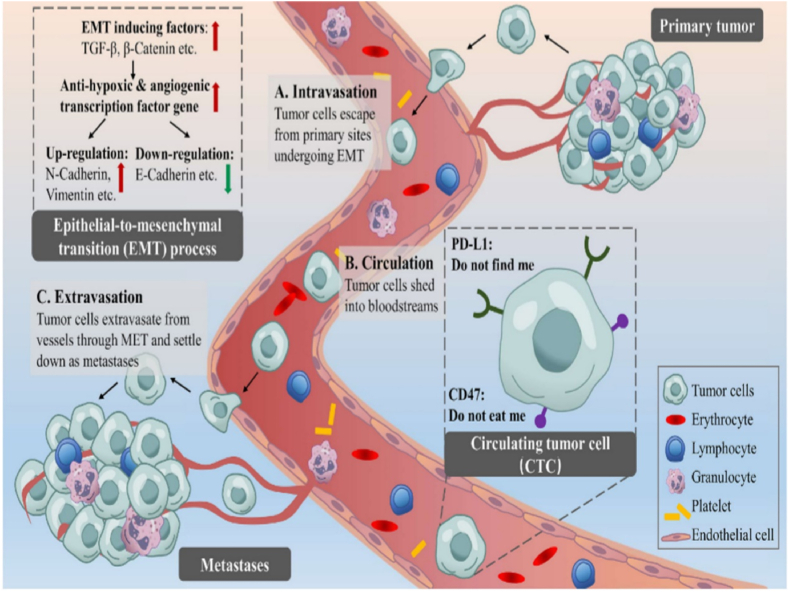
Liquid biopsy for the peripheral blood. Circulating tumor cells (CTCs) are the essential elements, widely believed to be the cornerstones of liquid biopsy. After undergoing the epithelial-to-mesenchymal transition (EMT) process, some CTCs shed from the primary tumor sites, travel through bloodstreams, and colonize into distant sites, contributing to regional or distant metastases [23].

In recent times, there has been a surge of optimism regarding circulating tumour biomarkers, commonly referred to as "liquid biopsies." Circulating biomarkers, particularly CTCs, have the potential to provide valuable biological insights from both main tumour and metastatic deposits [19]. CTCs, initially recognised by Thomas Ashworth in 1869, were characterised as neoplastic cells that are released from primary tumour locations and metastatic lesions into the bloodstream, potentially establishing themselves as metastases [20]. In 1889, Stephen Paget introduced the "seed and soil" hypothesis, which posits that CTCs can be likened to "seeds" that have a strong attraction to the particular environment, referred to as "soils" [21]. Scientists have shown that CTCs can accurately represent the diversity of tumours and offer valuable insights on the biological characteristics and genetic patterns of metastatic cascades [22]. Advancements in isolation and characterization techniques have made this possible. In recent decades, CTCs have provided a new opportunity for the treatment of OC, enabling early detection, classification of prognosis, real-time dynamic evaluation, and guiding treatment choices for individuals with OC [22]. In summary, CTCs, a liquid biopsy technology, demonstrate significant potential and offer numerous clinical applications ranging from laboratory settings to patient care (Fig. 2).

#### 1.1.1 CTC: physiologic characteristics and analysis

CTCs are malignant cells that are present in the peripheral circulation and can either infiltrate or be expelled from a primary or metastatic solid tumour location. Researchers have devised and verified a number of analytical techniques for the isolation of CTCs in ovarian cancer cases. These methods rely on different biological indicators, such as positive epithelial markers and negative hematopoietic markers, as well as physical characteristics, including size, density, deformability, electric charges, and invasive capacity [24]. The detection of CTCs in the bloodstream holds significant prognostic significance in the context of ovarian cancer. This is due to its ability to identify possible micrometastasis, pre-neoplastic lesions, tumour heterogeneity, and tumour evolution across time [22,25].

The technical difficulty in isolating CTCs from peripheral blood samples arises from their low concentration, which is estimated to be around 1 CTC per 1,000,000 circulating cells [26]. After being released from the main tumour, CTCs encounter various challenges in order to persist in the systemic vasculature and metastasize to remote organs [27]. Tumour cells that are lost from solid tumours frequently traverse the endothelium in order to get access to the bloodstream through the process known as epithelial-to-mesenchymal transition (EMT). EMT is a process in which epithelial cells undergo a phenotypic transition, resulting in the loss of polarity, shape, and cell markers, including the epithelial cell adhesion molecule (EpCAM). This metamorphosis allows the cells to acquire the migratory and invasive characteristics of mesenchymal cells [28].

After separating from a primary model V-2 carcinoma (derived from cottontail rabbit skin cancer) at a site that is 1 cm in size, every day about one million CTCs enter the bloodstream through dermal invasion. Typically, less than 1 % of these CTCs are able to spread to other parts of the body [29]. Apoptosis or necrosis is commonly observed in most CTCs as a result of significant environmental factors present in the bloodstream, including hunger, shear stress, and immunological identification [30]. Only a minority of CTCs are able to survive by increasing the production of growth factors, reducing the activity of death receptors, and expressing anti-apoptotic ligands [8]. CTCs must also elude the immune system's inherent defence mechanisms and avoid detection by natural killer (NK) cells. Tumour cells evade the activation of the antitumor response from NK cells and T cells by increasing the expression of the naturally existing programmed death ligand 1 (PD-L1), as proposed by the adaptive immune resistance theory. In addition, they exhibit resistance to phagocytosis by macrophages by upregulating CD47 [31,32].

At present, the CellSearch detection system holds the distinction of being the predominant isolation approach, having received approval from the US Food and Drug Administration (FDA). The immunoaffinity-based isolation approach employed by CellSearch is used to identify CTCs by detecting positive EpCAM expression [33].

Additional innovative methodologies encompass modified immunoaffinity-based approaches that simultaneously target multiple ligands, such as EpCAM, folate receptor alpha, and human epidermal growth factor receptor 2. Moreover, this repertoire also includes nanoparticles coupled with the antibody against Mucin 1 (MUC1) [34].

#### 1.1.2 CTCs heterogeneity and molecular makeup of CTCs

Similar to the primary tumour of ovarian cancer (OC), CTCs exhibit substantial heterogeneity and have distinct molecular compositions, both in terms of phenotype and morphology, which aids in the identification of several subpopulations of CTCs. CTCs may vary in their capacity to undergo EMT and inhibit apoptosis based on a set of molecular attributes. During the process of EMT, the Slug transcription factor usually enhances the production of vimentin. This, in turn, might raise the expression of Axl tyrosine kinase and subsequently cause alterations in the cytoskeleton of the migratory mesenchymal phenotype [21]. The intercellular connections of N-Cadherin adhesion ligands exhibit lower tightness compared to Cadherin 1 (CDH1) and epithelial Cadherin. This difference leads to a gradual loss of CAMs interacting with the extracellular matrix (ECM) and subsequent detachment from adjacent tumour cells [35].

In terms of morphological heterogeneity, in addition to individual CTCs, CTC clusters represent a rather uncommon classification consisting of 2–over 50 cells [36]. Several studies have asserted that clusters of CTCs have greater metastatic potential compared to individual CTCs. In their study conducted in 2016, Kevin et al. demonstrated that the metastasis of CTC clusters may be attributed to the involvement of keratin 14+ (K14+), a protein that plays a role in immune evasion, cell-matrix adhesion, and cell-cell adhesion [36]. Additionally, researchers observed that tumour cells within clusters exhibited anoikis and demonstrated a notable absence of apoptosis, maybe attributed to the persistent maintenance of survival signals mediated via cell junctions [37]. Further research is required to gain a comprehensive understanding of the heterogeneity and molecular composition of CTCs, with the aim of identifying potential strategies to inhibit the growth of ovarian cancer.

#### 1.1.3 Techniques for CTC test

The CTC diagnostic technique consists of a series of laboratory steps, including CTC enrichment, CTC identification, and further downstream analysis. Because of the scarcity of CTCs, the CTC enrichment technique is critical. Enrichment strategies utilising at least one physical and/or biological property of CTCs are currently employed in CTC platforms. After the enrichment process, we identify the CTCs using immunofluorescence or reverse-transcription PCR (RT-PCR). Furthermore, there have been advancements in the methodologies pertaining to downstream single-cell analysis.

##### 1.1.3.1 Biological techniques for CTC tests

Biological procedures widely employ the immunoaffinity approach for the enrichment of CTCs. The immunoaffinity approach has the potential to enhance the enrichment of CTCs through the utilisation of epithelial markers while simultaneously reducing the presence of blood cells by employing hematopoietic markers. The immunoaffinity technique for CTC enrichment was initially observed by Racila et al. [38]. Subsequently, the CellSearch® system, which was granted approval by the FDA in 2004, emerged as the pioneering immunomagnetic-based 10.13039/100007404CTC platform for the isolation and quantification of CTCs in metastatic cases of breast, colorectal, or prostate cancers [39]. The immunomagnetic method uses a magnetic field to effectively separate the CTCs, which are linked to antibody-magnetic bead complexes, from the other blood cells.

A biologic technology for enriching CTCs is the immunoaffinity-based microfluidic platform. It relies on microfluidic chips that have a nanostructure covered with a PEG-biotin-streptavidin layer [40]. Prior to analysis, the blood sample undergoes pre-treatment with biotinylated antibodies. The PEG-biotin-streptavidin layer on the microfluidic chip will bind to the target cells by interacting with the biotinylated antibody on the microvilli of CTCs. This binding occurs when the mixed-cell suspension flows over the chip.

EpCAM, an antigen associated with cancer, is commonly observed to be upregulated in cancer cells originating from epithelial tissues [41]. Anti-EpCAM antibodies are commonly used in immunoaffinity-based CTC procedures to enrich CTCs. However, EMT during cancer metastasis can decrease EpCAM expression and can vary depending on the cancer type. EMT is a crucial mechanism in cancer cell spread, causing cells to lose cell polarity and adhesion, transform into mesenchymal stem cells, and acquire migratory and invasive characteristics. Variability in EpCAM expression among cancer cells can significantly impact the capture effectiveness of immunoaffinity-based CTC platforms. EpCAM overexpression is prevalent in 73 % of epithelial ovarian cancer and as low as 55 % in serous adenocarcinoma of the ovary [42]. Combining both epithelial and mesenchymal antibodies can significantly enhance capture rates, mitigating the adverse effects of low EpCAM overexpression on CTC capture rates obtained using a single anti-EpCAM antibody [43,44].

##### 1.1.3.2 Physical (marker-independent) techniques

The physical methodologies employed in this study are predicated on the disparities in physical characteristics between CTCs and non-malignant blood cells. These disparities encompass variations in cell sizes, densities, electrical charges, and deformability. Numerous studies have emerged in recent times that explore the application of microfluidic-based physical techniques, including microfiltration, hydrodynamics, and dielectrophoresis, with the purpose of enriching CTC [45].

##### 1.1.3.3 Automation of CTC platforms

Over the course of recent decades, numerous methodologies have been devised to enhance and ascertain the characteristics of CTCs. Among these technologies, microfluidics-based CTC platforms have shown the potential for rapid advancement [46]. Nevertheless, the utilisation of these CTC platforms in clinical practice remains limited. Currently, the majority of CTC approaches continue to heavily depend on manual labour, leading to inconsistent test outcomes and limitations in conducting a greater volume of tests. As a result, automation of the laboratory process at CTC has emerged as a significant problem. Recently, there have been advancements in the development of automated systems for CTC detection. Aguilar-Avelar et al. developed a high-throughput automated microscopy technique for the analysis of CTCs that is both independent and reliable [47]. Ma et al. presented a study that showcased an automated immunoaffinity-based CTC platform capable of efficiently capturing SKBR3 breast cancer cell lines. This platform has been utilised for non-invasive foetal diagnosis [40].

#### 1.1.4 CTCs propagation in cell culture models

Developing cell cultures (in vitro) or growing them into xenografts (in vivo) to perform functional assessments is tough due to the rarity of CTCs. Nevertheless, significant endeavours have been made thus far to promote the growth of CTCs in several models, such as conventional two-dimensional (2D) cultures, CTCs-derived explants (CDXs), and three-dimensional (3D) organoids [48].

##### 1.1.4.1 2D CTCs culture models

According to current research, oncologists have identified several limitations associated with the traditional cultivation of tumour cells on a monolayer dish. These drawbacks include genotypic drift, loss of tumour heterogeneity, cross-contamination with other cell lines, and the need for in vitro adaptation [21]. As an example, a recent review found that common OC cell lines like A2780 and SKOV-3 lack important traits, including TP53 mutations and a lot of genomic instability. Consequently, the validation of research conducted using these cell lines is constrained [22]. A further investigation was carried out using the in vitro culture of enriched CTCs. The study showed that there was an upregulation of HER2 and CD24 expression, while the expression of CD44, CD68, CD45, and ALDH1 was reduced. This was achieved by GEA by PCR [49]. In a recent study, Yu et al. effectively generated six CTC lines using the CTC-iChip technology. These lines were then cultured in serum-free conditions for a duration exceeding six months, exhibiting tumorigenic characteristics in mice [50].

Although CTC lines have been successfully expanded in vitro in OC, it is important to acknowledge the limitations described earlier. These limitations may be attributed to the intrinsic drawbacks of 2D cultures, such as their adherence to plastic, absence of tumour microenvironment, and lack of in vitro spatial interactions [51]. Overall, 2D CTC lines do not accurately reflect the in vivo environment of OC, emphasising the need to create alternative culture models.

##### 1.1.4.2 CTCs-derived xenografts (CDXs)

Alternatively, oncologists constructed CTCs-derived xenografts (CDXs) by implanting CTCs orthotopically or subcutaneously into immunocompromised mice, in order to mimic original cancer conditions in an in vivo setting, with less genetic divergence and more tumor heterogeneity [52]. Recently, CDXs have been considered as a reliable in vivo translational research platform of various solid malignances, in order to provide insight into the mechanistic basis of cancer biology and test therapeutic strategies.

However, the feasibility of CDXs still remains challenged due to several caveats, including time-consuming, labor-intensive, ethically problematic, and high-cost [51]. Moreover, CDXs are not amenable for study on immunotherapy, due to the lack of immune system in immunocompromised mice [52].

##### 1.1.4.3 CTCs-derived organoids

In recent times, there has been a growing interest in the utilisation of CTC-derived organoids as a viable approach to address the pressing need for a favourable preclinical model centred around CTCs. In 2009, Sato and his colleagues achieved the development of advanced organoids by successfully creating a mini-gut culture system using small intestinal crypts from mice [53]. Organoids have been effectively generated from primary tumour lesions, metastatic locations, and dendritic CTCs [54]. Thus far, scientists have created organoids produced from OC CTCs using initial tumour cells and two essential external factors, namely the culture medium and extracellular matrix [55]. In the process of constructing organoids, researchers isolate a population of CTCs from the body fluid of patients with OC. Subsequently, the CTC pellet is seeded into the extracellular matrix, typically Matrigel, and supplemented with a combination of growth factors. This supplementation serves to enhance the viability of the culture by replacing paracrine and endocrine signals within the in vivo environment [55].

Previous studies have found that organoids produced from CTCs are suitable for genetic alterations and high-throughput screening assays. These organoids exhibit tumour heterogeneity and biological stability that closely resemble those of the original tumours [51]. CTC-derived organoids serve as dependable preclinical models that complement genetic testing by offering valuable insights into the medication response of individuals with OC. This has the potential to enhance clinical decision-making. While it may appear simpler to create organoids directly from tumour tissues, conventional tumour biopsy is invasive and not always feasible to acquire. This presents significant opportunities for CTCs as a more desirable source for cultivating organoids [54]. The CTCs-derived organoids model will have extensive applications in the future, spanning from laboratory settings to clinical settings.

#### 1.1.5 CTC test as a “real-time liquid biopsy”

The progress in automating CTC platforms and developing high-efficiency microfluidic chips has shown the potential for clinical use of CTC diagnostics. Numerous studies have examined the relationship between CTC counts and illness progression. Moreover, research on CTCs has progressively advanced from just counting the number of CTCs to conducting in-depth molecular examinations of the cells. These studies have also explored the use of CTCs as a "real-time liquid biopsy" to track the advancement and prognosis of the disease, as well as the development of tumour cells. The examination of single cells in CTCs provides valuable insights into the molecular features and tumour progression of the illness, thereby enhancing our understanding of the disease and facilitating informed decision-making in its management.

### 1.2 Circulating tumour DNA (ctDNA)

#### 1.2.1 A brief overview of ctDNA

ctDNA is becoming recognised as a highly promising and innovative alternative biomarker for the detection and monitoring of cancer, with both prognostic and diagnostic capabilities. The initial documentation of ctDNA in the bloodstream of individuals diagnosed with cancer dates back to the 1970s [56]. Subsequently, the identification of TP53 mutations in the bodily fluids of patients diagnosed with bladder cancer was accomplished [57]. cfDNA is released into the bloodstream during the processes of tumour apoptosis, necrosis, or active release. ctDNA is obtained from a portion of the overall cfDNA [58], and its half-life in the bloodstream is shorter than 2 h [59]. Brief DNA fragments, typically ranging from 150 to 200 base pairs, compose ctDNA. Because of ctDNA is a promising diagnostic technique due to its distinctive and circulating half-life. A collection of extensive investigations that examined various primary tumour types, including ovarian, bladder, and colorectal malignancies, as well as different phases, demonstrated a 6-log difference in the concentration of ctDNA [60]. Furthermore, it has been observed that ctDNA is present in more than 50 % of cancer patients, and it has exhibited significant associations with the molecular pathophysiology of solid tumours [61]. Furthermore, ctDNA has the potential to facilitate the observation of the entire tumour genome as opposed to a particular segment. Furthermore, the use of ctDNA analysis offers a noninvasive approach to acquiring tumour tissue by biopsy, enabling the systematic collection of samples to assess the temporal variations in both quantity and composition. The demonstration of evolutionary adaptability in response to platinum chemotherapy and poly ADP ribose polymerase (PARP) inhibitors using ctDNA analysis is significant. The detection of bridge reversion mutations resulting from these treatments in certain individuals can be readily identified within ctDNA, providing a valuable tool for cancer monitoring [62]. Presently, the proliferation of noninvasive cancer diagnostic testing has been facilitated by advancements in the analysis and isolation of ctDNA technologies [63]. In its entirety, ctDNA is increasingly becoming recognised as a viable clinical approach that may effectively capture both spatial and temporal variations in tumour heterogeneity (TH).

#### 1.2.2 Detection

Currently, many techniques, such as polymerase chain reaction (PCR)-based and next generation sequencing (NGS)-based procedures, have been created to detect the specific mutations associated with cancer in the ctDNA found in the bloodstream. PCR-based methodologies have effectively been utilised in the examination of ctDNA. Nevertheless, their utility is restricted to identifying particular predetermined mutations. Digital PCR (dPCR) or droplet dPCR (ddPCR), a third-generation PCR method, has demonstrated a remarkable specificity of 81 % and an exceptional sensitivity of 99 % for detecting a specific location in gynaecological tumours. This technique enables precise measurement of the amount of nucleic acids and can be used to analyse biological materials for specific mutations or wild-type sequences using fluorescent probes. On the other hand, Next-Generation Sequencing (NGS) enables the detection of genes with high sensitivity across many areas of the genome in a single test. It has been utilised for profiling DNA mutations and determining the burden of mutations in tumours. Alternative methods, such as whole-genome sequencing (WGS) and cancer-personalised profiling by deep sequencing (CAPP-Seq), utilise next-generation sequencing (NGS) to analyse ctDNA in gynaecological tumours [8,64,65]. These methods have a wide range of uses, including assessing tumour mutation burden, identifying epigenetic changes, and diagnosing or detecting resistance mutations. In gynaecological tumours, many approaches have been used to detect and analyse ctDNA with a high level of diagnostic sensitivity and specificity.

#### 1.2.3 Clinical application of ctDNA

The presence of ctDNA is a result of the release of tumour cells into the peripheral bloodstream, where it may be identified as a component of cfDNA [66]. ctDNA [67] provides a wealth of information regarding tumoral genetic variants, making it the leading method for liquid biopsy of tumours. Recent research has indicated that ctDNA analysis holds great potential for disease screening, early detection, monitoring disease development, evaluating prognosis, and monitoring therapy response [68]. For example, a mutation in PI3KA in ctDNA may have the ability to predict the response to the PI3K inhibitor alpelisib with nab-paclitaxel in metastatic breast cancer that is negative for HER2 (human epidermal growth factor receptor 2) [69]. HPV ctDNA has been identified as a reliable indicator for early diagnosis of relapse and residual illness in cervical cancer [70].

The examination of ctDNA has revealed that ctDNA may possess a greater capacity to reflect tumour heterogeneity compared to individual tumour biopsies. This is due to the prevailing belief that all cells contribute to the collective pool of ctDNA [71]. This method enables the evaluation of tumour genomes throughout the entire body using a single blood sample. This procedure can be repeated at regular intervals by collecting fresh blood as needed, facilitating tumour monitoring [72]. Furthermore, previous studies have demonstrated that the identification of ctDNA after surgery can serve as an indicator of tumour relapse, suggesting the presence of modest residual disease [73]. The identification of tumour-specific mutations holds considerable potential for the development of targeted anticancer treatments [74].

An inherent drawback of ctDNA is the extensive sequencing depth necessary to identify low levels of ctDNA, especially in the initial stages of cancer, as a result of dilution caused by cfDNA generated from normal, healthy cells. Furthermore, it should be noted that DNA analysis merely encompasses a fraction of tumour biology, leaving the remaining aspects to be evaluated by tumour biopsy techniques such as histology or the examination of alternative blood-borne liquid biopsy procedures. Elevated levels of plasma cfDNA have been observed in persons diagnosed with endometrial or ovarian malignancies in comparison to those who are not afflicted by these diseases [75].

At present, the absence of a distinct biomarker for the subsequent assessment of EC necessitates the reliance on imaging modalities for regular clinical surveillance. According to Pereira et al., it was hypothesized that ctDNA serves as a standalone prognostic indicator for the survival rate of individuals diagnosed with ovarian and endometrial malignancies. Consistent surveillance of ctDNA levels in individuals with gynaecological tumours can detect disease recurrence at an earlier stage compared to CT scans. Additionally, monitoring ctDNA levels following therapy can offer a novel indicator of patients' survival rates [76]. A recent study with a limited number of participants also showed that [77] ctDNA can identify the reoccurrence or advancement of EC around 2.5 months (1–8 months) earlier than traditional imaging methods. This study identified ctDNA in patients with stage I EC who later experienced a relapse. An increased level of ctDNA was indicative of disease advancement, as confirmed by imaging methods. Additionally, it can accurately indicate the response to radiotherapy in patients with recurrent EC who are undergoing chemotherapy. Nevertheless, there are limitations to conducting prospective real-world, large-sample studies. There is a scarcity of studies examining the relationship between ctDNA and EC recurrence and advancement, and the sample size is small. Therefore, additional research is necessary to address this gap.

Researchers have also investigated the use of cell-free ctDNA as a means of monitoring medication response and disease recurrence in patients with OC. A recent study investigated the potential of genome-wide copy number variations in ctDNA as new biomarkers in patients with high-grade serous ovarian cancer [78]. In a substantial majority of plasma samples, liquid biopsy analysis by shallow whole-genome sequencing revealed an increase in clonal areas, specifically 3q26.2 and 8q24.3. Moreover, ctDNA analysis demonstrated superior performance compared to CA-125 in predicting both clinical and radiological progression. This indicates that ctDNA analysis is valuable for monitoring the evolution of diseases after therapy and predicting illness relapse [78]. In the plasma of individuals with polycystic ovary syndrome (PCOS), it has been observed that BRCA1/2 reversion mutations are present in the ctDNA [79]. A study of ctDNA from PROC cases in a group of gynecologic cancer patients found changes in many genes, including EGFR, KRAS, TP53, PIK3CA, MYC, BRAF, MET, CCNE1, and CDK6. These changes accounted for 78 % of the cases. Furthermore, a greater frequency of ctDNA mutations was linked to poorer overall survival [80]. Patients with OC have identified TP53 mutations in their ctDNA samples [81]. Additionally, researchers have used bisulfite sequencing to detect CpG methylation in the peripheral blood DNA of OC patients treated with platinum-based drugs during their first relapse. Hence, the utilisation of blood-based DNA methylation as a new biomarker holds promise in the prediction of overall survival among patients with PROC [82].

### 1.3 Cell-free DNA (cfDNA)

#### 1.3.1 The origin and biological characteristics of cfDNA

Recent research has substantiated that while there is variability in cfDNA levels among individuals, cancer patients have elevated levels. Plasma samples from healthy individuals contain trace levels ranging from 1.8 to 118 ng/ml [83]. In contrast, cancer patients exhibit a range of 59–819 ng/mL. Moreover, previous studies have demonstrated that the percentage of plasma ctDNA among the total cfDNA ranges from 10 % to 90 % [84]. This plasma ctDNA might originate from the main tumour, metastases, and CTCs in individuals with cancer, thus encompassing the entirety of the tumour burden [85].

**Fig. 3:**
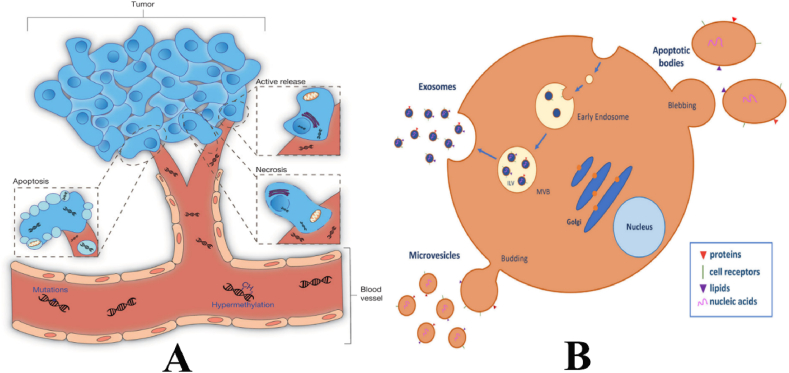
A. The origins of cell-free tumour DNA. Tumour cells can release DNA into the blood stream by three different mechanisms, such as apoptosis, necrosis and spontaneous active release, making tumour-related genetic and epigenetic alterations detectable in blood [92]. B. Extracellular vesicle (EV) biogenesis and secretion. EVs are classified into three major subtypes on the basis of biogenic and morphological properties: exosomes, microvesicles (MVs), and apoptotic bodies [5].

Several issues pertaining to cfDNA biology remain inadequately understood, including the cellular mechanism of DNA release, the clearance process from the bloodstream, and the biological ramifications of cfDNA. The prevailing notion that cfDNA originates from dying cells that discharge their material into circulation has gained significant attention. It is believed that cfDNA originates from apoptotic cells in people who are in good health. The fact that normal plasma samples predominantly contain 10.13039/100026054DNA fragments measuring 180 bp and larger, indicative of apoptotic cells, supports this. Fragments bigger than 2000 bp are not observed in these samples [86]. Apoptosis has been suggested as the potential source of cfDNA in cancer patients, as evidenced by multiple investigations indicating that their plasma and serum DNA exhibit a ladder pattern following electrophoresis, which closely resembles the pattern observed in apoptotic cells [84]. Nevertheless, the validity of this idea is subject to scrutiny due to the purported loss of apoptosis in cancer cells as well as the presence of a distinctive ladder pattern in actively released DNA [87]. Researchers have identified necrosis as a potential mechanism for the release of cancer DNA into the bloodstream. This is supported by the observation of plasma 10.13039/100026054DNA fragments larger than 10,000 base pairs, which are characteristic of cells undergoing necrosis [84]. Nevertheless, the concept is contradicted by the observed reduction in plasma DNA levels after radiation therapy [88]. If necrosis were the primary mechanism of DNA release by cancer cells, we would expect an initial elevation in cfDNA levels. Another frequently proposed hypothesis is the lysis of CTCs, although this is evidently not the primary mechanism for DNA release due to the extremely low occurrence of CTCs [89]. The presence of unlikely levels of CTCs would be required to account for the ctDNA concentrations observed in the plasma and serum of cancer patients [90]. Another potential and credible hypothesis is that cancer cells undergoing proliferation actively secrete DNA into the circulatory system, employing a mechanism similar to that reported in lymphocytes cultured in vitro [91]. However, the primary mechanism of DNA release remains undetermined, and it is highly probable that other mechanisms are involved (Fig. 3A).

#### 1.3.2 Methodologies in exploring cfDNA

Plasma germline cfDNA has a lower ratio compared to serum in blood collection, making it more appropriate for isolating ctDNA [93]. The expeditious execution of plasma separation is imperative due to the relatively brief half-life of ctDNA, which is approximately 2 h in conventional EDTA tubes [72]. Certain blood collection tubes, such as Streck, are designed with specific preservatives that enable the separation of plasma at a later stage [94]. The plasma that has been separated after centrifugation should be stored at a temperature of ℒ80 °C for future use.

The extraction of cfDNA and ctDNA is crucial for the subsequent assay. The approaches that are now accessible encompass phase isolation, spin columns based on silicon membranes, and isolation based on magnetic beads [95]. The extraction efficiency exhibits variability based on the employed methodologies and the quantities of plasma [96]. Novel methodologies have been developed to analyse cfDNA or ctDNA with exceptional sensitivity and specificity, both quantitatively and qualitatively [97]. These techniques include PCR strategies and NGS strategies.

PCR-based methods encompass a range of techniques, each possessing distinct characteristics. These include real-time PCR, co-amplification at lower denaturation temperatures (PCR), methylation-specific PCR, digital PCR or droplet digital PCR (ddPCR), BEAMing, and others [93]. Specifically designed to identify hotspot mutations, these PCR-based approaches are renowned for their efficiency and cost-effectiveness.

The assessment of genetic alterations can be conducted using NGS-based methods, including tagged-amplicon deep sequencing (TamSeq), the Safe Sequencing System (SafeSeqS), cancer personalised profiling by deep sequencing (CAPP-Seq), and targeted error correction sequencing (TEC-Seq), each with varying detection capacities [93]. The utilisation of whole genome sequencing (WGS) and whole exome sequencing (WES) technology enables the detection of previously unknown genetic modifications. NGS-based approaches have an advantage over PCR in their ability to detect copy number variants (CNVs) [93]. While these strategies are effective, they are also time-consuming and necessitate expert bioinformatics analysis.

#### 1.3.3 Clinical application of cfDNA

A considerable body of research has been conducted on cfDNA in individuals diagnosed with ovarian cancer in order to elucidate its clinical significance [98]. In order to achieve this objective, the researchers measured the overall concentration of cfDNA and/or the levels of circulating cell-free mitochondrial DNA (mtDNA) in certain instances. Additionally, they sought to identify various genetic and epigenetic modifications, including chromosomal abnormalities, specific tumour loss of heterozygosity (LOH), somatic gene mutations associated with cancer, and abnormal DNA methylation. Furthermore, Martignetti et al. [99] identified the FGFR2-FAM76A tumour-specific fusion in the cfDNA of a patient with advanced-stage serous epithelial ovarian cancer in a recent case study.

The initial investigations into circulating DNA in ovarian cancer aimed to measure the overall amount of cfDNA, as well as the amounts of nuclear and mitochondrial DNA individually, in patients' plasma or serum. A pioneering investigation on the utilisation of cfDNA in the screening of ovarian cancer sought to measure the presence of cfDNA in plasma by employing a real-time PCR assay for three reference genes. Additionally, the study tried to ascertain the quantity of genome equivalents (GE) by utilising a standard curve. According to the findings of Kamat et al. [100], the levels of cfDNA in advanced ovarian cancer samples were found to be higher in comparison to the control group. A recent study investigating the screening of ovarian cancer using the quantification of cfDNA revealed a statistically significant elevation in serum cfDNA levels among patients with advanced-stage ovarian cancer in comparison to those in the early stage (p < 0.01). Shao et al. [101] used receiver operating characteristic (ROC) curves and a branched DNA (bDNA) approach to quantify cfDNA and found a link between serum cfDNA levels and the prevalence of ovarian cancer.

The researchers observed that DNA methylation, as determined using clinical liquid biopsies, exhibits pharmacological reversibility and holds potential as a marker for preventive, diagnostic, and therapeutic purposes [102]. The therapeutic efficacy of hypomethylating drugs, such as decitabine or azacitidine, in reversing DNA methylation has been demonstrated in the treatment of haematological malignancies. The significance of reversible gene methylation as a potential therapeutic target is underscored by the limited number of large-scale clinical investigations conducted on female tumours [103]. Inhibition of the maintenance methyltransferase DNMT1 can easily reverse DNA hypermethylation [104]. The examination of methylation heterogeneity in various types of female tumours using liquid biopsy analysis, as well as the investigation of methylation regulation linked to sex hormone receptors, presents significant prospects for future research in the development of treatment strategies aimed at reversing DNA methylation.

The utilisation of liquid biopsy-based DNA methylation analysis has the potential to enhance the early diagnosis of cancer, hence potentially resulting in a significant decrease in mortality rates associated with cancer. Using liquid biopsies in clinical settings has shown growing success in improving detection methods that aim to find accurate molecular markers. This has helped us learn more about how cancer develops in women. Additionally, liquid biopsies have proven to be effective in executing such procedures. Conducting DNA methylation testing in high-risk female populations has the potential to improve individuals' quality of life by preventing disease and enabling early-stage therapies. The potential of liquid biopsy testing, which offers both high specificity and sensitivity at a reasonable cost, is encouraging in the current routine clinical situation. Future research should focus on investigating methylation liquid biopsies in large cohorts of clinical female oncology patients.

### 1.4 Circulating cell-free RNAs (cfRNAs)

Tumours undergo rapid turnover, leading to increased gene transcription and the release of large quantities of cfRNA, which includes mRNA and microRNA (miRNA), into the bloodstream [105]. MiRNAs are secreted by both normal and cancer cells into several bodily fluids, such as plasma, urine, vaginal secretion, and breast milk [106]. Within the circulatory system, messenger RNA (mRNA) and miRNA are effectively associated with distinct ribonucleoprotein complexes, high-density lipoproteins, platelets, or EVs such as exosomes. This arrangement serves to prevent degradation and enhance overall stability [106]. Numerous studies have posited the involvement of miRNAs in several biological processes, including cancer, cell differentiation, proliferation, angiogenesis suppression, metastasis, and death. Significantly, miRNAs exhibit accelerated synthesis and activation, accompanied by extended half-lives in comparison to mRNA and proteins. This characteristic renders miRNAs potentially more appropriate for the early detection of ovarian cancer [107]. Numerous studies have investigated the diagnostic, prognostic, and therapeutic capabilities of circulating miRNAs in the context of ovarian cancer.

The examination of RNA molecules is a relatively recent methodology in the field of cancer diagnostics, with the objective of uncovering abnormalities in gene expression and the occurrence of alternative splicing. Due to advancements in molecular characterization, there is significant potential to identify RNA indicators that can aid in clinical decision-making in colorectal cancer (CRC).

#### 1.4.1 Messenger RNA (mRNA)

Plasma samples contain protein-coding mRNA as well as short-non-coding RNAs and other nucleic acids [108]. Cell-free mRNAS (cfmRNA), similar to cf.miRNAS, are believed to be present in conjunction with extracellular vesicles as well as being present in the bloodstream [109]. Unlike cf.miRNA, cfmRNA analysis allows for the quantification of gene expression responsible for protein synthesis and the identification of tumor-specific mutational variations. However, the latter can also be achieved by the study of cfDNA, which exhibits greater stability and necessitates fewer specialised extraction techniques [109]. There is a scarcity of publications about the utilisation of cfmRNA compared to cf.miRNA due to its lower stability (prone to frequent degradation) and lower abundance in the bloodstream, which poses challenges in its detection and analysis [109].

The presence of MLH1 mRNA in blood samples shows potential as a biomarker for identifying and differentiating individuals with LS from those who are healthy. It has been predicted to have a sensitivity of up to 82 % and a specificity of up to 87 % [110]. Scientists have looked at the MMR genes and found sequence variants that can change gene expression directly by changing mRNA splicing, transcription levels, polyadenylation, and/or RNA stability. Around 30 % of documented MMR mutations interfere with the process of normal RNA splicing [111]. A comprehensive cohort study involving about 370 individuals diagnosed with LS revealed that 40 % of the patients possess an MLH1 mutation, with the predominant kind of mutation being a modification impacting a splice site [112].

#### 1.4.2 MicroRNA (miRNA)

MicroRNAs (miRNAs or miRs) are a class of small, non-coding RNA molecules that consist of around 22 nucleotides. These molecules play a critical role in the regulation of gene expression after transcription, mostly through gene silencing. Upregulation of miRNA results in decreased mRNA translation or destruction of its transcript by binding to several mRNA targets [113]. The dysregulation of miRNA expression in cancer is a significant factor that influences various cellular processes, including cell proliferation, differentiation, apoptosis, and stress response. This dysregulation is achieved through the upregulation of oncogenes and the downregulation of tumour suppressor genes [114]. A rising body of research has provided evidence for the regulatory role of miRNAs in the biology of breast and gynaecological cancer [115,116].

The isolation of cell-free miRNAs is a straightforward process using commercially available kits, and previous studies have demonstrated their exceptional stability, even in the presence of endogenous RNase activity [117]. This enables the isolation of sufficient quantities of miRNAs for high-quality sequencing. The most often employed analytical approach is NGS sequencing, which involves a reverse transcriptase step [118]. The stability of these entities can be attributed to their interaction with protein complexes or their encapsulation within extracellular vesicles [119]. They have been identified in many bodily fluids, such as urine and saliva, in addition to plasma [120]. A wide range of clinical applications for cf.miRNAs have been investigated in the context of cancer, encompassing early detection, disease surveillance [120], therapy response prediction, and disease prognosis [121]. Their research is limited by the fact that most miRNAs are present in both tumour and normal tissue, rather than being specific to malignancies. The analysis of cf.miRNA expression is contingent upon the relative levels, which can present challenges when the quantity of cf.miRNA released from tumours is minimal or in the early stages of disease.

Recent identification of cf.miRNA has led to largely exploratory research in this field. In the pursuit of enhanced circulating biomarkers, cf.miRNA has been assessed, and multiple laboratories have demonstrated that cf.miRNA analysis can differentiate between those who are healthy and those who are ill [119]. Specific microRNAs, namely miR-205 and let7f, have demonstrated significant diagnostic precision in the context of early-stage ovarian cancer. Notably, let7f has exhibited prognostic significance, as seen by its association with low levels, indicating an unfavourable prognosis [122].

Endometrial cancer research has also revealed varying amounts of cf.miRNA between healthy individuals and those affected. The study found that miR-15b, miR-27a, and miR-223 levels were different in people with endometrioid endometrial cancer compared to people who did not have the disease. This suggests that these genes may play a role in enhancing the detection of endometrial cancer; however, further exploration is needed [123].

Epithelial cancer frequently contains miRNA-21, also known as miR-21. It typically enhances the prevention of cell death in different types of cancerous tissues and cell lines, including breast cancer, ovarian cancer, and epithelial cancer. This is achieved by reducing the activity of tumour suppressors such as phosphatase and tensin homolog [124], as well as programmed cell death protein 4 [125]. The overexpression of miR-21 in the tumours of patients with breast cancer has been found to be correlated with advanced tumour stage, lymph node metastases, and worse survival outcomes [126]. In OC cell lines, miR-21 stimulates pathways that increase resistance to chemotherapy [127].

Unlike miR-21, let-7 members of the miRNA family are mostly known for their ability to decrease tumour growth by reducing the activity of the Harvey rat sarcoma viral oncogene homolog and the high mobility group AT hook 2 [128]. Nevertheless, research has yielded inconclusive findings for the specific constituent 7b. While several studies have indicated a positive correlation between elevated levels of let 7b in both serum and plasma and a favourable prognosis in cancer [129], a prior meta-analysis has shown that high-grade serous OC with elevated tissue expression of let 7b is related to worse survival rates [130]. The miRNA family miR-30, which suppresses tumour growth, has been found to promote apoptosis by inhibiting the activity of ubiquitin conjugating enzyme 9 and integrin β3 [131]. In British Columbia, miR30a hinders the movement and infiltration of cells [132], while the presence of miR30c in tissues is linked to advantages in endocrine therapy [133] and the regulation of processes related to resistance to chemotherapy [134]. It is worth mentioning that there is a significant increase in the expression levels of miR 30c and miR 30e in OC as compared to normal tissue. Nevertheless, both miRNAs have been found to be linked to a more favourable prognosis [135].

MiRNAs present in single-copy RNAs (sncRNAs) can aid in differentiating between high-grade and low-grade endometrioid tumours. In a prior investigation [136], which involved 36 patients with EC and 40 individuals without the disease, it was found that a substantial level of MiR-182 expression was strongly linked to the development of high-grade endometrial cancer. Furthermore, they determined the AUC values for MiR-200a, MiR-20b, MiR-200c, MiR-205, and MiR-182 to be 0.958. The sensitivity and specificity, as well as the positive and negative predictive values, were found to be 92 %, 89 %, 89 %, and 91 %, respectively. These results indicate that miRNA detection for endometrial cancer has a high level of diagnostic accuracy. In addition, a comprehensive analysis of 82 studies revealed that MiR could potentially serve as a prognostic indicator for the management of EC patients, particularly when it is associated with prognostic markers such as lymph node status, lymph node metastasis, and survival without recurrence [137].

#### 1.4.3 Long non-coding RNA (lncRNA)

lncRNAs represent an additional category of non-coding RNAs that have a role in the regulation of gene expression across several levels [138]. ncRNAs encompass both long and short ncRNAs. It is widely recognised that they might have a dual function as either a carcinogen or by impeding the advancement of tumours in gynecologic cancers. Certain academics also believe that ncRNAs possess significant promise as targets for personalised drug therapy [139]. This approach has the potential to mitigate the adverse effects associated with conventional medication and enable the development of unique targeted medicines for various patient subgroups.

Several studies have shown that circulating lncRNAs have the potential to be useful biomarkers for diagnosing and predicting the outcome of different types of malignancies. Furthermore, it has been observed that circulating lncRNAs have potential as biomarkers in several stages of the validation process, including pre-analytical, analytical, and post-analytical stages, for the purpose of detecting and treating cancer [140]. Small vesicles, protected by membranes, typically encapsulate lncRNAs. These vesicles can take the form of exosomes, microparticles, apoptotic bodies, or are fused with certain stabilising proteins. Subsequently, LncRNAs are actively released into different bodily fluids, including serum, saliva, and urine [141]. The median half-life of lncRNAs has been documented as 3.5 h, surpassing the half-life of numerous mRNAs [142]. Previously, researchers considered these entities to be "transcriptional noise." Nevertheless, current research has demonstrated their substantial involvement in the regulation of various crucial biological processes linked to cancer, such as carcinogenesis, cell proliferation, metastasis, and therapeutic responses [143]. Considerable research has been devoted to investigating the potential utilisation of lncRNAs as biomarkers for cancer. This has involved examining the associations between changes in lncRNA expression and various human clinical diseases, particularly those linked to cancer [140].

Researchers have found that the lncRNAs MALAT1, HOTAIR, H19, OVAL, CCAT1, and others exhibit carcinogenic effects in EC [144,145]. One of the genes that exhibits responsiveness to oestrogen in endometrial cancer is HOTAIR. When the level of expression of the gene increases, there is an observed increase in the rate of metastasis, accompanied by a drop in the overall survival rate [146]. The expression of OVAL is highly prevalent in type I EC, while its expression is relatively low in type II EC [145]. Consequently, OVAL has the potential to serve as a marker for the diagnostic classification of EC. CCAT1 has the ability to influence the proliferation of endometrial cancer cells as well as the activation of genes that control growth, thereby exerting an oncogenic function. Additional investigation is required in subsequent studies to examine the possibility of this target as a therapeutic intervention for EC [144]. Furthermore, there exists a strong correlation between the elevated expression of PCAT1 [147] and many factors such as FIGO stage, myometrial invasion, lymph node metastases, and reduced overall survival in individuals diagnosed with endometrial cancer. Consequently, this biomarker holds potential as a valuable tool for predicting the prognosis of patients diagnosed with endometrial cancer. A recent investigation has revealed that the lncRNA SRA plays a role in the proliferation, migration, and invasion of EC cells. This involvement is mediated by the upregulation of ELF4E-BP1 expression and the activation of Wnt/β-catenin signalling pathways. These findings suggest that SRA could potentially serve as a novel biomarker for predicting the recurrence and prognosis of endometrial cancer. Furthermore, SRA holds promise as a potential therapeutic target for this type of cancer [147].

#### 1.4.4 Circular RNA (circRNA)

circRNAs are non-coding nucleic acids that have a closed-loop structure and are functionally active. This structure renders them extremely resistant to the action of RNases [148]. Due to the recent identification of circRNAs, our comprehension of their role in tumour development remains unclear. Circular RNAs have been postulated to possess the ability to interact with microRNAs and proteins, hence facilitating the modulation of gene expression and splicing mechanisms [149].

The advent of high-throughput sequencing and microarray technology has led to a growing body of research indicating that circRNAs possess diagnostic or prognostic significance in several types of cancer [150]. circRNAs have varied expression patterns in tumours, exhibiting distinct clinical characteristics, indicating their potential prognostic significance. Multiple studies have substantiated the predictive significance of these factors in many types of cancer, such as ovarian cancer [149]. The poor prognosis of patients with OC was found to be associated with a decrease in circ-ITCH expression [151]. A decrease in the presence of hsa_circ_0078607 was found to be linked to an unfavourable prognosis, such as an advanced FIGO stage and elevated blood CA125 level [152]. So far, a number of studies have demonstrated the diagnostic significance of circular RNAs (circRNAs) in cancer through the use of lipid biopsies [153]. In squamous cervical carcinoma, the expression of circFoxO3a in the blood was reduced and found to be associated with tumour invasion, lymph node metastases, and a negative prognosis [154]. The use of microarray technology has been used to detect abnormal circRNA expression patterns in the plasma of individuals with breast cancer. It has been determined that hsa_circ_0001785 has the potential to serve as a valuable diagnostic biomarker in breast cancer [155].

circRNAs are plentiful and varied, with a half-life of more than 48 h, making them easier to detect [156]. The expression of circRNA exhibits variations across primary and metastatic sites and is believed to have a regulatory function in the context of ovarian cancer. According to a recent study, there exists an inverse relationship between the expression levels of circular RNAs and the activation of many signalling pathways implicated in cancer metastasis, such as NF-κB, PI3k, AKT, and TGF-β [157]. In their study, Hu et al. employed RT-qPCR to analyse a cohort of 83 patients with EOC in comparison to 166 benign or healthy controls. Their findings revealed a significant association between CircBNC2 and histological grade, serous subtype, and distant metastasis [158]. In a similar vein, it has been observed that lncRNAs play a role in the initial development, advancement, spread, and resistance to chemotherapy of recurrent ovarian cancer [159]. Despite the presence of emerging evidence indicating a potential link between varying levels of lncRNAs such as H19, LSINCT5, XIST, CCAT2, HOTAIR, AB073614, and ANRIL and the clinical progression or treatment response of ovarian cancer, there is still a need for a comprehensive understanding of the diagnostic sensitivity and specificity of lncRNAs [160,161]. Currently, there have been no lncRNAs that have received approval for clinical use. Additional study is necessary to determine the most clinically significant candidates that possess cancer-enriched or particular characteristics in ovarian cancer.

### 1.5 Extracellular vesicles (EVs)

#### 1.5.1 Extracellular vesicles

Tumor-derived EVs are lipid bilayer-bound vesicles that originate from cells with a diameter ranging from 30 to 2000 nm. Originally perceived as mere membrane detritus, these entities have subsequently demonstrated significant involvement in intracellular communication through the transportation of proteins, lipids, and nucleic acids [162]. The classification has three primary categories, namely exosomes, microvesicles, and apoptotic bodies. The endolysosomal route generates exosomes, while the plasma membrane budding process creates microvesicles. The apoptotic process, which shares the same nomenclature, forms apoptotic bodies through the regulated demise of cells. EVs obtained from various pathways exhibit variations in size and content, including the presence of specific surface marks. Nevertheless, the presence of diverse surface markers can facilitate the enrichment of these subtypes through the process of marker selection (Fig. 3B).

EVs exhibit a wide range of functions, encompassing the modulation of immunological responses, tissue regeneration, and hemostasis [163]. Exosomes have a main function in presenting antigens and monitoring and activating the immune system [164]. Additionally, both exosomes and microvesicles facilitate genetic communication between cells by transporting nucleic acids [165]. Due to their pivotal role in controlling cellular activities, it is unsurprising that EVs may have a significant role in the development of cancer and various other disorders. The involvement of these entities in the progression of tumours is multifaceted, encompassing the facilitation of growth and angiogenesis, the facilitation of immune evasion, and the establishment of pre-metastatic environments [166].

Due to the direct involvement of EVs in various crucial stages of tumour genesis and development, there is an increasing interest in regulating their impact. This encompasses the suppression of their synthesis, extracellular liberation or absorption, and the obstruction of certain extracellular EV constituents [163]. EVs encompass a diverse array of circulating constituents, such as DNA, mRNA, microRNA, and other non-coding RNAs. Consequently, the investigation of any individual component within EVs poses challenges [165]. Indeed, it is plausible that they could serve as a potential origin for these constituents in plasma analysis. However, it is important to acknowledge that the composition of circulating nucleic acids varies from that observed in EVs, and the various subtypes of EVs also exhibit distinct compositions [167].

EVs face challenges due to their inherent variability and complexity. The research findings derived from an examination of EVs from a single cell type may vary depending on the conditions of the cell culture, variations in purification techniques, or the methodologies employed to characterise the EVs [168]. Ultrafugation has traditionally been considered the most effective method for purifying exosomes. However, concerns about the poor activity of the collected EVs have prompted the development of other approaches such as liquid chromatography or marker precipitation-based separation [169].

The production of EVs by tumour cells is widely recognised. Due to their involvement in cellular communication, intricate composition, and diverse contributions to tumour growth and development, EVs possess a substantial reservoir of biological information pertaining to the tumours they originate from. Consequently, they have been assessed for several functions in cancer, such as prognostic biomarkers [170]. Disease monitoring can be conducted using EVs present in blood plasma as well as several other body fluids, including urine and saliva [171]. Plasma EVs have the potential to differentiate between people who are impacted and those who are not, and they may also differentiate between individuals with early and late stages of the disease. Additionally, they may have an increased presence of immunosuppressive proteins such as PD-L1, PD-1, and CTLA4. Researchers have linked elevated levels of PD to increased disease activity and an advanced stage of the disease. Researchers have demonstrated the correlation between disease response and the monitoring of protein levels in EVs, suggesting that EVs could potentially play a role in assessing the response to therapy. This implies that EVs have the potential to serve as an indicator of disease advancement and forecast the presence of advanced disease. Furthermore, EVs may have implications for future treatment decisions, such as the stratification of immune checkpoint inhibitors [170]. An increasing body of research suggests that EVs may play a significant role in the early diagnosis of diseases, owing to their easy accessibility and the presence of bioactive substances within them [172].

#### 1.5.2 Exosomes

**Fig. 4:**
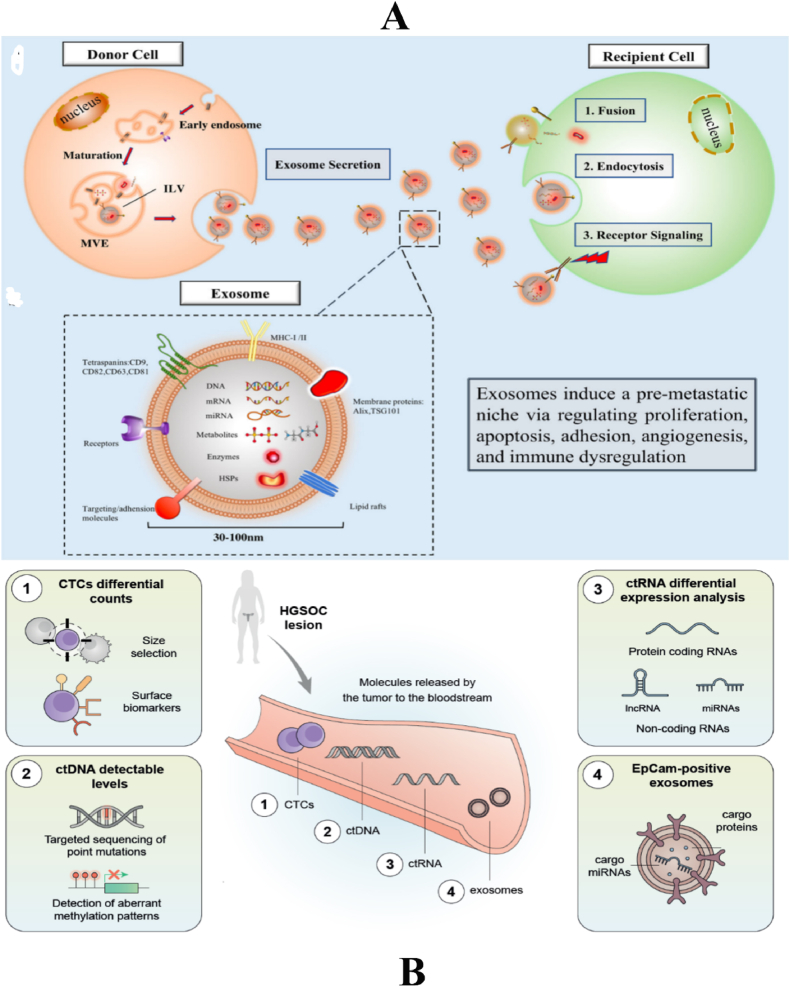
A. a. The biogenesis of exosomes and the mechanisms involved in intercellular communication. The exosome is an intraluminal vesicle (ILV) formed by the inward budding of the endosomal membrane during the maturation of the multivesicular endosomes (MVEs) and then secreted after fusing with the cell surface. Uptake by recipient cells occurs via a three step processes: 1. membrane fusion with target cells; 2. endocytosis; 3. activation of surface receptors and signaling. b. Schematic diagram of the exosome. The exosome is a disk-shaped membranous vesicle with a diameter 30–100 nm, and carries a parental cell cargo including lipids, metabolites, proteins, nucleic acids (DNA fragments, mRNA, miRNA, etc.) [173]. B. Liquid biopsies rely on isolating CTCs, ctDNA, ctRNAs, or exosomes released by tumours that enter the bloodstream. Different analytical techniques support the identification of putative biomarkers for the early diagnosis of HGSOC [174].

Exosomes are a kind of membrane-bound particle with dimensions ranging from 30 to 150 nm. These particles possess the ability to transport many forms of cargo, including proteins, lipids, genetic material, and other substances. Exosomes facilitate intercellular communication and exert influence on the functionality of receiving cells. These findings demonstrate promising research opportunities in the field of medicinal delivery vehicles. Recent times have witnessed notable advancements in the field of exosome research. Exosomes, an underexplored mode of intercellular communication, play a crucial role as carriers for the absorption, transportation, and release of many substances, including biomarkers and therapeutic targets. The utilisation of therapeutic exosomes for patient assistance is an innovative and possibly advantageous area of technology and therapeutic focus (Fig. 4A). We can effectively employ existing technologies to identify valuable diagnostic or prognostic indicators in these individuals.

##### 1.5.2.1 Structure and function of exosomes

The hypothesis of the presence of EVs was first proposed in 1946 and subsequently validated in 1967 [175]. EVs have been categorised into different categories according to their size, specific surface properties, formation process, and composition, mostly consisting of apoptotic bodies, microvesicles, and exosomes. Exosomes, as the most extensively studied and utilised variety of extracellular vesicles, possess the most theoretical and practical significance among the three. Therefore, we commonly refer to extracellular vesicles as exosomes, and in scholarly literature and everyday correspondence, we often use the term "exosomes" (exo) interchangeably with EVs.

Exosomes, a specific form of EVs, were initially identified by Bonucci and Anderson in the late 1960s [176]. The particles in question are extracellular vesicles characterised by a cell-like morphology. They typically exhibit a diameter ranging from 30 to 150 nm and possess a density of 1.13–1.19 g/mL. Consequently, they can be classified as the smallest nanoscale extracellular vesicles [177]. Endocytic cells generate exosomes, vesicles with a lipid bilayer membrane architecture. These vesicles undergo intracellular invagination to form multivesicular bodies, which then fuse with the plasma membrane for release.

The International Society of Extracellular Vesicles (ISEV) has published the Minimal Information for Studies of Extracellular Vesicles (MISEV2018), which suggests the use of nomenclature that denotes physical attributes of EVs. These attributes include size (small EVs (sEVs) < 100 nm or < 200 nm; medium/large EVs (m/lEVs) > 200 nm), density (low, middle, high), biochemical composition (e.g., CD63 + EVs), or descriptions of conditions or cellular origin (e.g., podocyte EVs) [178].

Almost all cell types produce exosomes under both normal and abnormal circumstances. Initially, exosomes were believed to be waste particles that removed unwanted cellular components. Recent studies have provided evidence that they have significant functions in facilitating intercellular communication by facilitating the transfer of nucleic acids as well as specific sets of proteins and lipids [179].

Exosomes contain a diverse array of components, including genetic material such as DNA, coding or noncoding RNA, proteins, lipids, and metabolites [180]. The size and payload of exosomes can exhibit variation, even when they originate from a single cell. Exosomes have lately been found to have significant involvement in various physiological and pathological processes, such as cell growth, cell death, the formation of new blood vessels, inflammatory pathways, the development of tumours, and the repair and restoration of tissues. Exosomes possess the potential to serve as biomarkers for the detection of their widespread release by numerous cells and diverse composition and functionalities. The utilisation of exosomal miRNAs as innovative diagnostic and prognostic biomarkers is a growing trend in the field of liquid biopsy [181].

##### 1.5.2.2 Exosomes and gynaecological malignancies

Exosomes are known to have a significant impact on intercellular communication. In the context of tumours, exosomes originating from tumours have been identified as agents that contribute to several aspects of tumour development and progression, including metastatic dissemination, increased angiogenesis, and treatment resistance. This is achieved through the regulation of stromal cells and the tumour microenvironment (TME). Tumour exosomes consist of three main components: DNA, RNA, and protein [182]. Researchers are currently studying the biogenesis, processes of carcinogenesis, development, and treatment of tumour exosomes, as well as the discovery of biomarkers in cancer [183].

### 1.6 Tumor-educated platelets (TEPs)

The involvement of tumour-educated platelets (TEPs) is significant in both local and systemic reactions to tumour proliferation. Platelets typically exhibit an absence of nuclei; however, they may possess leftover mRNA and miRNA that originate from their megakaryocyte ancestors or are acquired through intercellular interactions within the bloodstream. Platelet education refers to the process by which macromolecules from cancer cells are transferred and sequestered into platelets [184]. Specific splicing events of pre-messenger RNAs (pre-mRNAs) in circulating platelets can be induced by external stimuli in the cancer microenvironment, such as signals from stromal and immune cells [185]. The main benefits of TEPs include their abundant presence, straightforward isolation, and the production of high-quality RNA that can be processed based on external signals. Hence, TEPs exhibit a dynamic mRNA repertoire characterised by distinct splice events in response to external stimuli, as well as the direct uptake of spliced circulating mRNA. This characteristic renders TEPs potentially valuable for diagnostic purposes in the context of ovarian cancer. The diagnostic potential of TEPs was initially investigated by Best et al. by mRNA sequencing in individuals diagnosed with different types of malignancies [186]. The study revealed that TEPs have a remarkable ability to differentiate cancer patients from healthy controls, with an accuracy rate of up to 96 %. Additionally, TEPs were able to accurately identify the primary tumour location with an accuracy rate of 71 %. In a subsequent study, Piek et al. reached the conclusion that TEPs have the capability to distinguish early-stage ovarian cancer from benign diseases with an accuracy rate of 80 % [187]. A current clinical investigation (NCT04022863) aims to enhance these findings by investigating the precision of TEPs and ctDNA in identifying the characteristics of ovarian tumours and providing insights into their diagnostic capabilities [188]. Giannakeas et al. conducted a recent retrospective cohort study to investigate the correlation between thrombocytosis, defined as a platelet count exceeding 450∗10^9^/L, and the occurrence of cancer. The researchers conducted a study on 53,339 adults between the ages of 40 and 75 who experienced thrombocytosis, had a normal platelet count in the past 2 years, and had no history of cancer. They then calculated the probability of developing cancer over a 10-year period of follow-up [189]. The researchers indicated that the relative risk (RR) for ovarian cancer was highest at 2 years (RR = 7.11; 95 % CI, 5.59–9.03) and much greater at 6 months (RR = 23.33; 95 % CI, 15.73–34.61). There is potential for the development of a blood-based technique for cancer diagnosis in the future, wherein TEP profiling is combined with complementing ctDNA/CTC analysis and platelet quantification.

### 1.7 Metabolic biomarker detection

Metabolomics, the most recent discipline among the 'omics' sciences, enables the detection and characterization of all the metabolites (molecules <1 kDa) present in a biological system. It has the ability to provide a comprehensive approach to clinical medicine while also enhancing illness diagnostics and our understanding of pathological mechanisms [190]. Metabolites serve as significant indicators of physiological or pathological conditions, offering valuable insights for identifying disease biomarkers and enhancing our understanding of their development and advancement [191]. Metabolomics has the potential to serve as a powerful tool for early disease diagnosis as well as a predictor of therapy response and survival. In recent years, there has been a growing use of metabolome analysis in medical investigations for the purpose of detecting biomarkers and diagnosing diseases [192].

Various techniques have been employed in recent years to isolate and measure different components of the metabolome. It is important to note that no single analytical platform can fully capture all the information about metabolites in a sample. Mass spectrometry (MS) is a versatile and highly sensitive technique that may be employed to analyse and measure a wide variety of substances in a biological sample, even when the amounts of metabolites vary greatly [193]. The efficiency, sensitivity, and reproducibility of targeted and untargeted analyses have been significantly enhanced by recent advancements in MS and chromatography [194]. Metabolomics necessitates the effective separation of the substances to be analysed. Chemical separation techniques like GC, LC, or capillary electrophoresis can all be combined with MS detection. Thus, the use of hyphenated analytical techniques such as liquid chromatography (LC)-MS, gas chromatography (GC)-MS, capillary electrophoresis (CE)-MS, and matrix-aided laser desorption ionisation-MS frequently results in improved coverage of analytes in the metabolome [195]. The utilisation of these techniques can yield precise and clinically valuable diagnostic capacity for the treatment of disorders at the metabolic level [196]. Metabolomics using mass spectrometry (MS) will facilitate the identification of new biomarkers, perhaps enabling the early identification of different diseases.

The field of metabolic diagnosis shows potential but encounters obstacles due to the suitability of biospecimens and the low reliability of analytical methods. Ruimin Wang et al. [197] propose a diagnostic technique that combines dried serum spots (DSS) with nanoparticle-enhanced laser desorption/ionisation mass spectrometry (NPELDI MS). Our method enables the rapid and accurate diagnosis of several types of cancer in a matter of minutes. It is cost-effective, environmentally safe, and provides results comparable to those obtained from serum samples. Additionally, the protocol is designed to be user-friendly. Based on our evaluation, introducing this tool in underdeveloped areas has the potential to decrease the estimated percentage of undiagnosed cases of colorectal cancer from 84.30 % to 29.20 %, gastric cancer from 77.57 % to 57.22 %, and pancreatic cancer from 34.56 % to 9.30 %. This would result in an overall reduction ranging from 20.35 % to 55.10 %. This study offers valuable insights on optimising metabolic diagnostics for improved sustainability and optimum health benefits.

Epithelial ovarian cancer (EOC) is a multifactorial process characterised by changes in metabolic pathways. There is a strong need for a highly efficient screening method for EOC to enhance predictive outcomes; however, it has not yet been developed. The study conducted by Congcong Pei et al. [198] focuses on the development of a composite material consisting of a concave octahedron structure made of Mn2 O3 and (Co,Mn)2 O4 (MO/CMO). This composite possesses a heterojunction, a rough surface, a hollow interior, and sharp corners. Its purpose is to be used in laser desorption/ionisation mass spectrometry (LDI-MS) to record the metabolic patterns of ovarian tumours. The MO/CMO composites exhibit synergistic physical effects that result in heightened light absorption, favoured charge transfer, amplified photothermal conversion, and targeted entrapment of small molecules. The MO/CMO exhibits approximately 2–5 times greater signal enhancement compared to mono- or dual-enhancement alternatives and approximately 10–48 times greater enhancement compared to commercially available solutions. MO/CMO-assisted LDI-MS is used to uncover the serum metabolic fingerprints of ovarian tumours. This method allows for direct serum detection without any treatment and achieves a high level of reproducibility. In addition, the use of machine learning to analyse metabolic fingerprints enables the differentiation of malignant ovarian tumours from benign controls with a high level of accuracy, as indicated by the area under the curve value of 0.987. Ultimately, a total of seven metabolites that are linked to the advancement of ovarian tumours are examined as prospective biomarkers. The concept provides a framework for developing advanced techniques to diagnose MS with high accuracy. It also promotes the development of nanomaterial-based platforms for precise diagnosis scenarios.

## 2 Liquid biopsy in gynaecological tumours

The three most prevalent gynecologic malignancies are OC, CC, and EC, which make a significant contribution to the overall cancer burden worldwide. Ovarian cancer is the leading cause of death associated with gynaecological malignancies. The 5-year survival rate of OC is dismal due to the fact that over 70 % of OC patients are diagnosed at an advanced stage and have fast recurrence following initial treatment [199]. Experts predicted that cervical cancer, the fourth most prevalent malignancy among women, will cause approximately 604,000 new cases and 342,000 deaths worldwide in 2020. CC is a malignancy that arises from cells located at the junction of cervical squamous cell carcinomas. Infection with a strain of human papillomavirus (HPV) known for its high carcinogenicity is considered a required but insufficient factor in the development of CC [200]. Endometrial carcinoma is the second most common cancer in the female reproductive system and the sixth most common cancer among women [176]. Gynaecological cancer is prevalent in high-income nations, and its occurrence is on the rise worldwide. The survival rate of patients with gynaecological cancer is higher when the disease is detected, and the efficacy of clinical treatment and the subsequent improvement in prognosis are also noteworthy. There is an urgent demand for biomarkers that are sensitive and specific and can be used in peripheral areas.

Currently, the identification and assessment of malignancies depend on imaging investigations and tissue biopsy. The detection of tiny lesions or limited residual tumours is subject to constraints even with the most modern imaging systems. People continue to widely regard tissue biopsy as the preferred method for establishing a conclusive diagnosis of questionable tissue. However, it can be intrusive and certain lesions may be located in inaccessible areas. Moreover, tissue biopsy has the potential to promote tumour metastasis in instances of gynecologic malignancies. Hence, the identification of novel prognostic biomarkers is critical in order to improve gynecologic cancer management.

The "liquid biopsy" is gaining significance as a crucial tool in the pursuit of precision oncology's objective to revolutionise cancer care, rapidly transitioning from the research environment to the clinical context [201]. Primarily, the efficacy of this technology has been directed towards addressing challenges associated with advanced disease in those who have already been diagnosed and are undergoing active treatment. According to several studies [202,203], liquid biopsies are employed after the initial treatment to identify the existence of minimal residual disease, forecast the likelihood of disease recurrence, evaluate the development of drug resistance, and offer recommendations for customised salvage treatment. In stark contrast, the progress made in the field of early cancer diagnosis is currently constrained and characterised by varying levels of effectiveness [204]. An important limitation mentioned is the inherent scarcity of detecting ctDNA molecules and CTCs in tiny and early-stage tumours [205]. In addition, the identification of ctDNA or CTCs using liquid biopsy does not yield conclusive information regarding the nature or source of the tumour.

### 2.1 Ovarian cancer (OC)

**Fig. 5:**
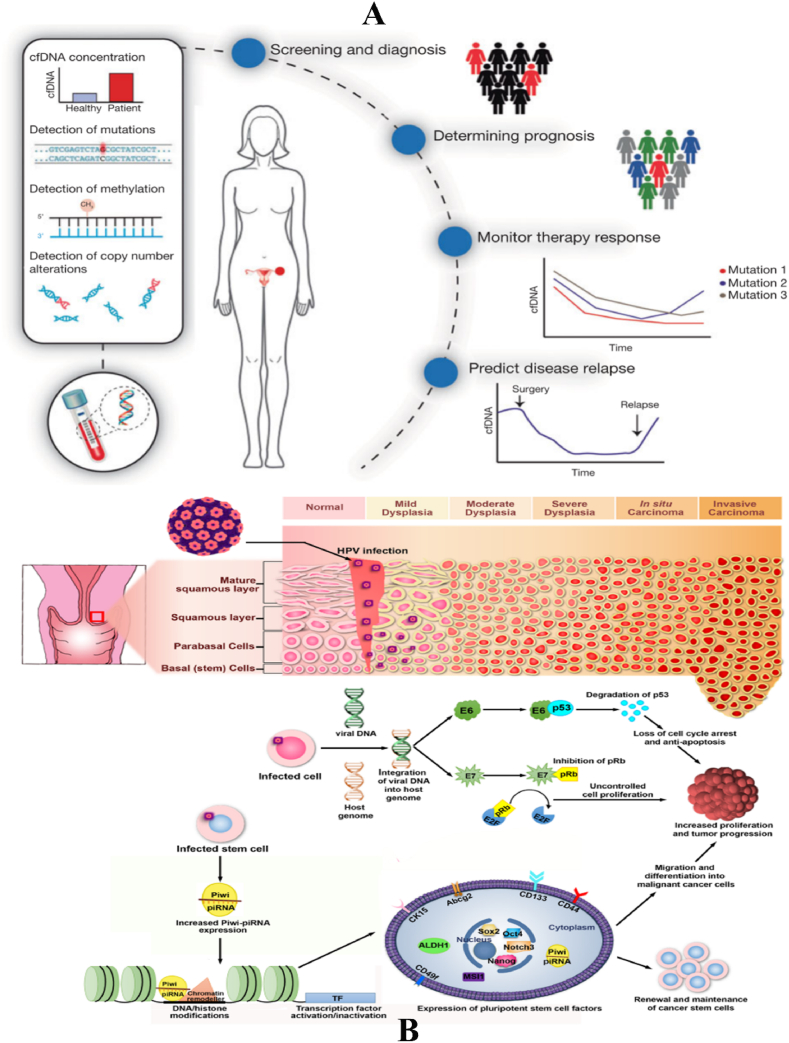
A. Clinical applications of liquid biopsies in ovarian cancer patients. cell-free tumour DNA (cfDNA) can be obtained from peripheral blood of ovarian cancer patients [92]. B. Schematic representation of HPV-associated cervical tumori-genesis [214].

According to the American Cancer Society, ovarian cancer has been documented as having the greatest mortality rate among gynecologic malignancies, with about 50 % of new cases being reported annually [206]. The majority of individuals diagnosed with ovarian cancer are often at an advanced stage, characterised by the dissemination of tumours. The treatment strategy for the disease may vary depending on its stage, encompassing surgical intervention, chemotherapy, radiation therapy, hormone therapy, or targeted therapy. These interventions aim to reduce the size and eradicate the primary tumour while also inhibiting the spread of the disease to other parts of the body [207]. The responsiveness of patients to typical treatment regimens varies clinically due to the intricate nature of the disease, namely the cellular and molecular heterogeneities of the tumour. Hence, the ability to forecast the effectiveness of treatment during the initial phases of therapy might improve the precision of patients' decision-making on the selection of suitable treatment protocols, with a special emphasis on chemotherapy [207]. The utilisation of liquid biopsy approaches has demonstrated enhanced patient outcomes in diverse cancer types, such as lung and breast cancer, through personalised methods. However, when it comes to ovarian cancer, the selection of an appropriate approach to determine the appropriate treatment for the individual patient remains a subject of contention [208]. Further research is necessary to identify the specific biomarkers, such as the level of CA125 or HE4, that correlate with patient response to chemotherapies, in order to address this issue. Nevertheless, the precision and efficacy of these indicators in forecasting the response to chemotherapy vary among patients with diverse epidemiological and clinical characteristics [209]. Genomic profiling of tissue biopsy offers a momentary depiction of the ever-changing characteristics of tumour data and reveals the genomic makeup of the tumour at the moment of diagnosis [210]. Moreover, these problems are notably evident in patients who exhibit resistance to therapy or in the subsequent follow-up of the patient [211]. In recent times, there has been a growing interest in the identification and characterization of cancer-derived components, including CTC, exosomes, and ctDNA, commonly referred to as liquid biopsy (Fig. 4B) [212]. This development has introduced a novel approach to patient classification and personalised treatment [210]. Among these options, the identification of ctDNA tumour-specific mutations exhibits significant potential in patient selection and precision medicine. Additionally, it can be proposed as a prognostic determinant for predicting treatment response in various tumour types, such as lung, breast, colorectal, and melanoma cancers [212]. Previous literature reviews have examined the technological issues associated with the detection and isolation of CTC in conjunction with ctDNA as a diagnostic indicator for ovarian cancer (Fig. 5A) [213].

#### 2.1.1 The diagnostic value of LB in ovarian cancer

CA-125 is the primary marker utilised in the diagnostic assessment of EOC, serving several purposes such as evaluating therapy effectiveness and monitoring the disease progression in patients with OC [215]. Nevertheless, CA-125 has been observed to exhibit elevation in benign tumours as well as in several medical situations, including endometriosis, follicular cysts, pregnancy, and infection [216]. The diagnostic capabilities and classification of ovarian masses as benign or malignant are limited. In conclusion, relying solely on CA-125 to diagnose the specificity of this approach is suboptimal.

The diagnostic biomarker HE4 has recently gained recognition as a potential alternative to CA-125 in the diagnosis and monitoring of OC therapy effectiveness. A gene located on chromosome 20q12–13.1 encodes the protein HE4, which is N-glycosylated. The presence of HE4 has been observed in various benign gynaecological conditions, including ovarian cysts, uterine fibroids, endometriosis, endometrial polyps, and other ovarian cancers such as endometrial and cervical cancer. However, it is important to note that there are notable differences in the expression levels of HE4 between these gynaecological disorders and ovarian cancer [217]. In addition, HE4 has demonstrated superior sensitivity and specificity compared to CA-125 [218]. The integration of CA-125 and HE4 analysis enhances the diagnostic precision of ovarian cancer. Therefore, the assessment of serum HE4 levels can serve as a valuable tool in distinguishing between benign gynecologic conditions and ovarian cancer [219].

Recent years have seen a significant amount of research on the role of cfDNA in the timely identification and assessment of ovarian cancer. The sensitivity and specificity of cfDNA detection surpassed those of conventional tumour markers, indicating that the diagnostic efficacy can be enhanced through the simultaneous detection of these biomarkers.

The most prevalent mutation in high-grade serous ovarian cancer is the TP53 mutation, which constitutes about 95 % of somatic mutations [220]. Previous studies have documented the identification of TP53 mutations in cfDNA and ctDNA [221]. The research findings indicated the presence of identical TP53 mutations in both ovarian cancer tissues and corresponding blood samples. Certain individuals diagnosed with ovarian cancer, particularly those who have progressed to advanced stages of the disease, have plasma containing tumor-derived DNA mutations. Hence, the identification of TP53 mutations in cfDNA and ctDNA has the potential to aid in the classification of ovarian cancer and ascertain the malignant stage of the disease. Nevertheless, diagnostic efficacy has not been documented; varying methodologies and detection techniques yield disparate outcomes. Testing the sensitivity and specificity of diagnosing ovarian cancer is necessary. In the meantime, it is imperative to ascertain the suitability of additional mutations associated with ovarian cancer for diagnostic purposes. The investigation of gene mutations has significant promise. DNA methylation alterations have been identified as an initial occurrence in the development of tumours [222]. The potential utility of circulating DNA methylation as an early diagnostic marker for ovarian cancer has been suggested in previous studies. Both tissues and plasma samples associated with ovarian cancer showed multiple alterations in methylation patterns.

In addition, it has been observed that qualitative detection of DNA methylation exhibits superior diagnostic value when compared to quantitative detection [223]. Therefore, the presence of aberrant methylation in cfDNA and ctDNA can be utilised for the early detection of ovarian cancer, offering promising opportunities for clinical implementation. However, there is a distinction between the sensitivity and sensitivity of diagnosis. Examining the factors contributing to the variations is beneficial for enhancing the effectiveness of diagnosis; it is necessary to do additional research and validate the diagnostic significance of cfDNA methylation.

Chromosome instability is a significant indicator that can identify ovarian cancer. Despite the limited number of papers on chromosomal instability, initial investigations have indicated its utility in ovarian cancer detection and potential for application in clinical research. Vanderstichele et al. [224] did a study that showed chromosomal instability levels were significantly higher in cfDNA samples from people who had been diagnosed with ovarian cancer compared to both healthy controls and people who did not have cancer. The method for finding cfDNA had an AUC of 0.94, a specificity of 99.6 %, and a sensitivity that was 2–5 times higher than that of CA125 and the malignant index risk. This was especially true for cases of high-grade serous ovarian cancer. Therefore, chromosomal instability in cfDNA exhibits potential as a diagnostic tool for ovarian cancer, demonstrating both high sensitivity and specificity.

The CTC detection rate was reported in seven studies utilising the CellSearch® system, but the sensitivity and specificity were provided in only one study. The CellSearch® system exhibits a relatively low overall detection rate for patients with OVCA, ranging from 14.4 % to 26 % [225]. The detection rate of up to 60 % in stage II-IV OVCA was reported exclusively by Liu et al. [226]. The studies utilising the AdnaTest system, a platform for detecting CTC that integrates immunomagnetic technology with RT-PCR technology, also demonstrated a rather poor detection rate ranging from 14 % to 30 % [227]. The system's inherent limitations may account for the low detection rates. One possible explanation for this phenomenon is that hematologic spread may not be the primary mode of metastasis in EOC cases. Consequently, a limited number of CTCs may not be detected in the patient's peripheral blood, particularly during the initial phase of the disease [228].

Increasing evidence suggests that approaches for identifying CTCs can offer an early diagnosis of patients with ovarian cancer. For example, Pearl et al. utilised a novel CAM platform to identify CTCs. Their findings revealed a positive predictive value (PPV) of 77.8 % in detecting patients with stage I/II EOC, according to the Federation International of FIGO. Additionally, the PPV for detecting all stages of EOC was 97.3 %, surpassing the conventional CA-125, which had PPV values of 61.6 % and 92.1 %, respectively [229]. Similarly, a separate research team discovered that only 2.56 % of healthy female participants, who had no prior cancer, tested positive for CTCs. In contrast, 24.5 % of patients with EOC and FIGO stages II–IV showed positive results. This was determined using RT-PCR, which detected the gene expression of 11 potential CTC markers (PPIC, GPX8, CDH3, TUSC3, COL3A1, LAMB1, MAM, ESRP2, AGR2, BAIAP2L1, and TFF1) [230]. Researchers came up with the MetaCell method, which uses both fluorescence microscopy to look at the cytomorphology of cells and a panel of markers for gene expression analysis (EPCAM, WT1, KRT7/18/19, and MUC1/16) to more accurately find OC. This approach is based on a significant expression difference (p < 0.02) [231].

Lili Ge et al. [232] have identified the plasma circRNAs hsa_circ_0003972 and hsa_circ_0007288, as well as the combination of hsa_circ_0003972 and hsa_circ_0007288 (circ-COMBO) and circCOMBO and CA125, as potential novel circulating biomarkers for the detection of ovarian cancer. Conversely, the amalgamation of circCOMBO and CA125 exhibited the most superior diagnostic efficacy. The decreased plasma concentration of hsa_circ_0007288 has the potential to function as a biomarker for lymph node metastases in ovarian cancer. Additionally, it is worth noting that hsa_circ_0003972 and hsa_circ_0007288 have the potential to serve as therapeutic targets for ovarian cancer.

Kalle Savolainen et al. [233] conducted a study to evaluate the viability of liquid biopsies for the purpose of miRNA expression profiling in HGSOC. Notably, urine, plasma, and tissue samples collected from the same patients with ovarian cancer can identify the three members of the miR-200 family. The study of tumour tissue and plasma has the potential to differentiate between malignant and normal ovarian samples. Furthermore, a significant association was identified between the expression of miR-200 in the urine and plasma of individuals diagnosed with ovarian cancer, although no such link was found in people with benign tumours. This preliminary study showed that plasma and urine might be useful as liquid biopsies for studying miRNA biomarkers in HGSOC. However, further research is required to substantiate the present results in larger patient cohorts, particularly those with early-stage disease.

The found EV protein biomarkers exhibit potential for early identification of ovarian cancer through screening. The utilisation of a panel of biomarkers is deemed more advantageous and dependable for the early detection of OC and the screening of individuals at high risk, such as women with BRCA1 or BRCA2 mutations, first-degree relatives of OC patients, or those with a history of early breast cancer, due to the presence of tumour heterogeneity. Furthermore, the panel of biomarkers can be utilised to differentiate between low-grade OC and high-grade instances, thereby enabling the prediction of prognosis for OC patients and facilitating the selection of an optimal treatment. Furthermore, we can employ the compilation of biomarkers in the longitudinal assessment of therapeutic efficacy, which includes chemotherapy, immunotherapy, or combination therapy. In the field of exosome isolation and high-throughput screening of clinical samples, a number of multiplexed analytic platforms have been recently developed [234]. The United States has recently introduced ExoDx® Prostate (IntelliScore), a novel urine exosomal RNA-based test, for clinical application as a Laboratory Developed Test (LDT). The utilisation of a 3-gene expression panel enabled the detection of individuals with high-grade prostate cancer who had increased levels of the biomarker PSA [235]. The aforementioned platforms and assays serve as valuable instruments for accelerating exosome-related research and facilitating its clinical application in the detection of ovarian cancer.

Ovarian cancer is characterised by its inclination to invade the peritoneal cavity through the ascites, which allows it to effectively affect different organs inside the compartment. Starting from the initial phases, the ascites is composed of dislodged tumour cells, diverse immune cells, mesothelial cells, and tumour-associated exosomes. Exosomes have been successfully extracted from the ascites [236] and serum [237] of individuals diagnosed with ovarian cancer. Crucially, these exosomes possess distinct protein signatures that are specific to ovarian cancer. Small GTPases like Rab proteins, annexin proteins, tetraspanins like CD9, CD82, CD63, and CD81, heat shock proteins like Hsp90 and Hsc70, antigens like MHC I and II, Nanog, and enzymes like phosphate isomerase, peroxiredoxin, aldehyde reductase, and fatty acid synthase are some of these. The exosomal protein cargo not only exposes an underlying malignancy but also promotes the advancement of metastases in ovarian tumours. An instance of Nanog can be observed as a transcription regulator that plays a role in the proliferation of tumour cells and the self-renewal of cancer stem cells [238]. Exosomes obtained from the ascites of high-grade serous ovarian cancer have a notably higher level of Nanog expression in comparison to benign peritoneal fluid [239]. Studies on Nanog deletion have demonstrated a reduction in the migration and invasion of ovarian cancer cells [240].

Researchers have acknowledged the potential of salivary mRNA biomarkers and serum carcinoembryonic antigen (CEA) as liquid biopsy techniques for the identification of many types of malignancies. However, existing tests typically rely on a single category of biomarkers, limiting their ability to distinguish cancer patients from those without the disease due to inadequate sensitivity and specificity. This study aimed to assess the efficacy of a combined method involving CEA and salivary mRNA biomarkers in distinguishing between ovarian cancer patients and healthy controls. They structured their study into two distinct phases: a preliminary phase that focused on identifying and assessing various biomarkers, and a subsequent phase that independently verified the suitability of the chosen biomarkers. They enlisted a cohort of 140 individuals with ovarian cancer and 140 individuals without the disease during the initial stage of the study. They assessed the concentration of CEA in blood and five mRNA biomarkers in saliva (namely AGPAT1, B2M, BASP1, IER3, and IL1b). They then constructed a machine-learning model to differentiate between patients with ovarian cancer and healthy individuals. They identified a novel panel of biomarkers that demonstrated a high level of sensitivity (89.3 %) and specificity (82.9 %) in distinguishing between individuals with ovarian cancer and those without the disease. Subsequently, they utilised this set of indicators in separate validation research comprising 60 individuals diagnosed with ovarian cancer and 60 individuals without the disease. In the validation phase, the ovarian cancer patients were well distinguished from healthy controls, achieving a sensitivity of 85.0 % and a specificity of 88.3 % [241].

#### 2.1.2 The monitering value of LB in ovarian cancer response to therapy

While the majority of ovarian cancer patients experience full remission following primary debulking surgery and further chemotherapy, a significant proportion of individuals, up to 70 %, experience recurrence as a result of chemoresistance. The primary factor contributing to medication resistance and treatment failure in ovarian cancer has been suggested to be intra-tumour heterogeneity [242]. The term "intra-tumor heterogeneity" refers to the genetic differences that occur within a lesion as a result of the evolutionary changes in cancer cells during the complex carcinogenesis process. The transformation of a single malignant cell into a diverse tumour mass is influenced by the tumour microenvironment and the ability to adapt to different external selection pressures, such as avoiding apoptosis, promoting self-replication, and achieving replicative immortality. Subclones have the potential to undergo sequential linear development and expansion, or alternatively, they may exhibit branched trajectories by undergoing further divergence during their evolutionary trajectory [243]. The molecular characterization of all subclones of ovarian cancer is of utmost importance in order to make informed decisions regarding targeted therapy and to detect the development of acquired resistance in tumour cell clones over a period of time. In the context of treatment, liquid biopsy has the potential to provide a more comprehensive investigation of tumour heterogeneity and enable longitudinal monitoring of tumour progression.

CA125, a commonly employed marker for treatment and follow-up, exhibited suboptimal performance in clinical settings [244]. On the other hand, it has been observed that cfDNA and ctDNA may have a significant impact on indicating the effectiveness of treatment in cancer patients. The study demonstrated a substantial correlation between the levels of cfDNA and the burden of tumours. The cfDNA levels grew in tandem with the tumour burden. Put simply, the levels of cfDNA rose in cancer patients and declined following successful therapy. The fluctuations in cfDNA levels in cancer patients can serve as a dynamic indicator of ovarian cancer's growth and advancement. There is a statistically significant link between changes in cfDNA levels and treatment response. However, no correlation was found between cfDNA levels and CA15-3 and CA19-9 [245]. In addition, the utilisation of ctDNA can be employed for the dynamic assessment of treatment response [246], as the concentration of ctDNA remains undetectable beyond a six-month period following the initiation of therapy. It indicated that patients may exhibit a favourable response to treatment. Hence, cfDNA and ctDNA have the potential to function as significant biomarkers for tracking the advancement of diseases and evaluating the effectiveness of treatments.

Scientists have proposed a novel medication that specifically targets CTCs to prevent the spread of cancer cells, recognising their crucial role in OC metastasis. Phipps et al. developed an implantable shunt device that utilises the molecular mechanisms of CTC extravasation. This device comprises a microtube that is adorned with tumour necrosis factor-related apoptosis-inducing ligand (TRAIL) and E-selectin. Its purpose is to facilitate the rolling of CTCs and subsequently induce apoptosis [247]. Additionally, Manuel and his colleagues showed that epithelial markers (CK19 and MUC1), genes linked to stem markers (CD44 and CD24), and mesenchymal markers (CXCR4 and TIMP1) identified CTCs in patients with OC that had spread beyond the peritoneal region. Notably, the expression of TIMP1 was found to enhance cancer growth, indicating its significant potential as a target for therapeutic intervention in OC treatment [248]. Furthermore, Wan et al. (year) demonstrated that the activation of natural killer (NK) and CD8^+^ T cells through state changes may be responsible for the enhanced effectiveness of PD-L1 immune therapies in high-grade serous ovarian cancer (HGSC). This was achieved by employing single-cell RNA sequencing, transcriptional, and immune functional profiling techniques on organoids that were co-cultured with immune cells [249].

#### 2.1.3 Recurrence and metastasis

Ongoing research is investigating the clinical use of liquid biopsy to detect microscopic residual disease after primary debulking surgery. We do this to ascertain the prognosis, forecast survival outcomes, and identify disease recurrence at an earlier phase. From a therapeutic perspective, the use of liquid biopsy can assist in the identification of individuals who are more susceptible to relapse, allowing for the exploration of alternative management strategies and the potential inclusion of these patients in clinical trials.

While the majority of ovarian cancer patients exhibit a favourable response to treatment, advanced cases of ovarian cancer often have a recurrence within a span of 1–2 years. The patients' age, histological type, tumour stage, and other characteristics are interconnected. Ovarian cancer exhibits a high propensity for metastasis, with around 70 % of malignant tumours progressing to the pelvic and abdominal organs. The assessment of recurrence and metastasis mostly depends on the utilisation of CA125 and CT scans. However, it's crucial to acknowledge the limitations of CA125 and CT scans in providing dynamic and timely monitoring of ovarian cancer patients after recurrence. Furthermore, the detection of metastatic lesions has limitations. Nevertheless, the utilisation of cfDNA shows great potential in the surveillance of ovarian cancer patients' recurrence and metastasis. During the process of tumour recurrence, there was a subsequent increase in the levels of PIK3A-H1047R in cfDNA, which exhibited a significant connection with metastasis [250]. In brief, the assessment of cfDNA and ctDNA levels is valuable for the surveillance of tumour metastasis and recurrence. Additionally, the examination of gene mutations and methylation alterations in cfDNA and ctDNA has considerable importance in understanding tumour formation and progression. The observation of cfDNA and ctDNA alterations is indicative of the presence of ovarian cancer.

Moreover, there is additional data substantiating the involvement of ctDNA in identifying recurring illnesses. Recurrence can occur in up to 85 % of people with EOC after receiving their first treatment. Recurrence of EOC, commonly regarded as incurable, significantly limits the longevity of patients. Traditional recurrence markers, such as CA-125, and imaging modalities like CT and PET scans are commonly employed [251]. Recent research has indicated that the quantification of ctDNA has the potential to enhance the identification of relapse in comparison to conventional imaging methods and CA-125 [252]. Parkinson et al. conducted a study on TP53 mutations in the blood ctDNA of patients with relapsed HGSOC. They found that ctDNA was present at a level of at least 20 amplifiable copies/mL of plasma in almost all relapsed patients with a disease volume greater than 32- cm^3^ [253]. Minato et al. similarly identified ctDNA via droplet digital PCR in all patients who had experienced recurrent EOC, while they found no ctDNA in those who had not experienced a recurrence. In the majority of instances, the presence of ctDNA was observed prior to the indication of recurrence by CA-125 levels [254]. The results align with the research conducted by Pereira et al., which found that the average time it takes for ctDNA to predict recurrence compared to CT imaging is 7 months [255]. Hence, ctDNA possesses the capacity to serve as an early detection tool for the recurrence of EOC.

A further crucial aspect of cancer care involves the assessment of the necessity for additional treatment subsequent to achieving optimal tumour debulking. The prevailing approach to determining the need for supplementary adjuvant systemic treatment often relies on the disease stage and certain risk factors. Nevertheless, the absence of a dependable biomarker for identifying MRD or micro-metastasis sometimes complicates decision-making and might result in either insufficient or excessive therapy of the disease [256]. Identifying and eradicating MRD in patients with EOC continues to be a significant obstacle in the field of gynecologic oncology. The CTCs test is believed to have the potential to serve as a very sensitive and specific indicator for detecting MRD, which is now undetectable by existing biomarkers or the most advanced imaging techniques.

Tomoko Noguchi et al. [257] showed that CAPP-Seq can effectively be used to molecularly profile and monitor gene alterations in advanced ovarian cancer patients using NAC. Furthermore, the authors propose that liquid biopsy-based identification of the TP53 mutation and bTMB could potentially serve as novel biomarkers for assessing NAC response. The results obtained using liquid biopsy have the potential to facilitate MRD identification and evaluation following treatment. Despite the considerable amount of research undertaken on the genetic profile of gynaecological cancers utilising DNA extracted from tumour tissue, obtaining enough samples through tumour biopsy or surgical resection is typically challenging, especially for advanced or recurring ovarian cancer. Liquid biopsy is a less invasive procedure that enables convenient serial assessments and has the potential to accurately depict tumour heterogeneity at real-time intervals. The utilisation of CAPP-Seq for genetic profiling of ctDNA in gynaecological tumours holds potential for enhancing the development of personalised treatment algorithms and enabling real-time therapy monitoring.

#### 2.1.4 Resistance to chemotherapy

Patients often develop and advance resistance to chemotherapy, and the use of cfDNA or ctDNA in the treatment of chemotherapy-resistant ovarian cancer has a significant impact. The use of bevacizumab has been demonstrated by Steffensen et al. [258] as a valuable intervention in the management of multi-resistance epithelial ovarian cancer. The levels of cfDNA, acting as an assistive marker, can guide treatment. The presence of BRCA1/2 mutations in the ctDNA of ovarian cancer patients was observed, and these patients showed a positive response to targeted therapy with PARP1 inhibitors [259]. The investigation of BRCA1/2 mutations is a significant advancement and offers a more comprehensive understanding of the reaction to chemotherapy. However, observations indicate a significant prevalence of clinically acquired treatment resistance associated with reversion mutations. A sequencing study revealed the presence of BRCA1/BRCA2 reversion mutations in cfDNA in 21 % of ovarian cancer patients who were resistant to therapy [260]. They have observed a correlation between the acquisition of BRCA1/2 reversion mutations and resistance to therapy, suggesting that these mutations may have potential utility in predicting the chemotherapy response of ovarian cancer and subsequently informing treatment strategies for this disease. Nevertheless, the precise process remains ambiguous. Additional research is required to investigate and confirm the significance of BRCA1/BRCA2 reversion mutations in ovarian cancer.

Whether it's platinum-based chemotherapy or targeted therapy like PARP-inhibitors, treatment can put selective pressure on cancer cells that can cause them to change their genes in ways that help them evolve. Resistance to therapy, specifically platinum/PARP inhibitors, was found to be related to reversion mutations of BRCA1/2 in the plasma of patients with ovarian cancer [261]. These findings, taken together, demonstrate the ability of ctDNA to identify the occurrence of escape mutations and the reversal of BRCA1/2 mutations. Consistent surveillance using a liquid biopsy could facilitate the timely identification of resistance and the selection of other medications. Additionally, it has the potential to facilitate personalised combinatorial therapy, including chemotherapies, targeted therapies, or immunotherapy, which can effectively address distinct oncogenic factors or mitigate the development of resistance.

Platinum-based medications serve as the fundamental component of systemic therapy for OC. Platinum resistance is associated with the disease's continued presence or premature recurrence, as well as the treatment's ineffectiveness. The characterization of CTC has the potential to be a novel approach for evaluating potential resistance to platinum. The research conducted by Kuhlmann et al. and Chebouti et al. has shown that the existence of ERCC1-positive CTCs is linked to the resistance of ovarian cancer to platinum and is also associated with a negative prognosis [262]. Obermayr et al. have demonstrated that individuals diagnosed with platinum-resistant OVCA exhibit a higher prevalence of detectable CTCs expressing the Cyclophilin C gene in comparison to patients with platinum-sensitive OVCA [263]. A study conducted by Lee et al. showed a positive correlation between the identification of CTC clusters and platinum resistance [264].

Recent research suggests that EVs may link to the underlying mechanisms of chemotherapy resistance, which remains a significant challenge in the treatment of OC. Previous studies have provided evidence that EVs generated by OC cells treated with cisplatin have the ability to enhance resistance to chemotherapy in bystander cells through various signalling pathways [265]. Multiple studies have concentrated on specific chemicals within EVs. For instance, Yin et al. conducted a study that revealed a potential association between Annexin A3 in EVs released by OC cells and platinum resistance. Additionally, the researchers observed that the levels of Annexin A3 in the sera of patients with platinum resistance were higher compared to those with platinum sensitivity. Furthermore, the study indicated that a higher concentration of Annexin A3 in the serum was indicative of a poorer prognosis [266].

#### 2.1.5 The prognostic value of cfDNA/ctDNA in ovarian cancer

The overall prognosis for patients with ovarian cancer was unfavourable. Despite significant advancements in surgery and chemotherapy, the survival rate of individuals with ovarian cancer did not significantly improve. Research has shown that patients with advanced ovarian cancer have a significantly lower 5-year survival rate compared to those with early ovarian cancer. Hence, the identification of tumour markers is crucial in order to evaluate the prognosis of individuals diagnosed with ovarian cancer. The prognostic evaluation of ovarian cancer can be enhanced through the utilisation of quantitative analysis of cfDNA and ctDNA. When the concentrations of cfDNA surpass a specific threshold, there is an elevated likelihood of mortality, which is associated with a reduced survival rate among individuals diagnosed with ovarian cancer [267]. Researchers observed a correlation between the concentration of RAB25 in cfDNA and both overall survival and progression-free survival. Low levels of RAB25 were found to be a significant predictor of improved progression-free survival (PFS) and overall survival (OS) in epithelial ovarian cancer [268]. In patients with chemoresistant ovarian cancer, CfDNA also demonstrated predictive significance. Patients exhibiting elevated levels of cfDNA experienced worse PFS and OS [269]. As a result, monitoring cfDNA levels can facilitate the adjustment of therapy regimens and allow for the observation of ovarian cancer patients' conditions. The identification of genetic alterations in cfDNA and ctDNA holds significant implications for the prognostic assessment of ovarian cancer. 68 % of patients with stage 1 or 2 ovarian cancer had somatic mutations in their plasma. As the tumour stage progressed, the proportion of mutant alleles in ctDNA also increased. Patients with elevated ctDNA levels experienced worse PFS and OS [270]. Approximately 33 % of individuals diagnosed with ovarian cancer exhibit tumour-specific TP53 mutations in their plasma, resulting in a decreased survival rate. The presence of tumour DNA in the bloodstream was found to be a significant predictor of poor survival in a multivariate study. Patients diagnosed with serous ovarian cancer and expressing TP53 antibodies exhibited a low overall survival rate [271]. The TP53 mutation in the ctDNA of patients with high-grade serous ovarian cancer is linked to the stage of the disease. The presence of a significant TP53 mutant allele percentage in ctDNA three months after chemotherapy indicated a poor progression [272]. Compared to CA125, the predictive effect was more pronounced. Therefore, the identification of the TP53 mutation in cfDNA or ctDNA holds significant therapeutic value in assessing the prognosis of ovarian cancer. Furthermore, the examination of the TP53 mutation in the plasma DNA might ascertain the extent of malignant ovarian cancer and prove beneficial for postoperative monitoring [273]. Ovarian mucinous carcinoma exhibited a notably elevated prevalence of the KRAS mutation, which was found to be correlated with unfavourable overall survival outcomes [274]. The meta-analysis unequivocally confirmed the existence of the KRAS mutation in epithelial ovarian cancer. Furthermore, the KRAS mutation in cfDNA was found to be linked not only to a negative OS but also to a negative PFS [275]. The identification of the KRAS mutation in cfDNA has been advantageous in predicting the prognosis of individuals diagnosed with ovarian cancer. The PI3CA and KRAS mutations in cfDNA from ovarian clear cell carcinoma were identified by the researchers through the use of ddPCR. The findings revealed that individuals exhibiting elevated levels of PIK3CA-H1047R and KRAS-G12D exhibited a reduced PFS [276]. The fluctuations of two indices demonstrated greater sensitivity and speed than CA125. Therefore, the evaluation of mutation status can yield valuable insights into the prognosis of individuals diagnosed with ovarian cancer.

The utilisation of molecular characterization or genetic research techniques on CTCs has the potential to yield additional insights as prognostic indicators. In their study, Gonzalez et al. employed multiparametric mass cytometry (CyTOF) to conduct a comprehensive analysis of the phenotypic characteristics of individual cells in high-grade serous OC. Their findings revealed that patients with a negative prognosis exhibited a greater tendency to co-express vimentin, HE4, and cMyc cells [277]. Additional research has suggested that the adverse prognostic effect of CTC may be attributed to certain characteristics linked to treatment resistance, such as the existence of ERCC1, the cyclophilin C gene, Twist, or PI3Ka [262]. In a recent study conducted by Yang et al., it was found that both the counts of CTC and mesenchymal CTC were independent variables for recurrence in 152 patients with EOC [278].

Kuhlmann and colleagues utilised the AdnaTest technology (QIAGEN, Germany) to gather CTCs. They observed a substantial correlation between OC outcomes and ERCC1, a gene implicated in DNA repair and platinum resistance, which was expressed in CTCs [279]. The p-values for PFS and OS were 0.009 and 0.026, respectively. A study has also verified that patients with platinum resistance are more prone to having Cyclophilin C gene (PPIC) positive CTCs, which may suggest unfavourable outcomes for ovarian cancer [230]. Given the growing body of evidence that highlights the significant involvement of PD-L1 in the tumour microenvironment, scholars have also conducted investigations on PD-L1 using liquid biopsies. Their findings indicate a notable correlation between elevated PD-L1 levels in CTCs and a decreased 5-year OS (p = 0.003) and PFS (p = 0.019) [174]. Furthermore, a correlation has been identified by researchers between chemo-resistance and a distinct set of genes (MRP1–10, MDR1, RRM1/2, ERCC1) that have potential as therapeutic targets for patients with chemo-resistant ovarian cancer [28]. So, learning more about the link between CTCs and how they can be used in real life should help doctors use a less invasive liquid biopsy technique to actively track how cancer is spreading and how well treatment is working.

At this point, there isn't enough information to support the clinical uses of other liquid biopsy components, like cell-free miRNAs and exosomes, in terms of how well they can predict what will happen. The dispute in the field of cell-free miRNA studies has been caused by several limiting constraints, such as the absence of standardised experimental protocols, inconsistent normalisation processes, and insufficiently powered sample numbers for statistical analysis [280]. The prognostic significance of cell-free miRNAs, specifically the miR-200 family, including miR-200a, miR-200b, and miR-200c, has been substantiated by multiple studies [280]. Zhang et al. conducted a study including 40 patients diagnosed with EOC, whereby they employed Western blot analysis and enzyme-linked immunosorbent assays (ELISA) to examine exosomal protein markers [281]. Patients with EOC who had high levels of fibrinogen gamma chain (FGG) or lipopolysaccharide binding protein (LBP) mRNA expression had a poorer prognosis and shorter PFS and OS, according to the study. Specifically, the study demonstrated that FGG had a hazard ratio (HR) of 0.79 for OS and 0.77 for PFS, while LBP had an OS HR of 0.81 for OS and 0.77 for PFS [281]. Due to the presence of several tumour-derived constituents, EVs exhibit potential as a comprehensive prognostic biomarker, offering insights into both the tumour itself and its surrounding milieu. Despite this, it is hard to come to a firm conclusion because there isn't a single, accepted way to separate cell-free miRNA and EVs, and existing studies use small sample sizes. Consequently, further validation in larger cohorts is necessary.

### 2.2 Cervical cancer (CC)

Cervical cancer is a preventable form of cancer that poses a significant risk to women's lives. This particular form of cancer ranks as the second most prevalent among women, particularly in countries with lower and lower-middle income levels [282]. Based on the specific cells damaged, we can classify cervical carcinoma into two distinct forms: squamous cell carcinoma and adenocarcinoma. Squamous cell carcinomas originate in the parenchymal cells that line the inferior aspect of the cervix, whereas the glandular cells that line the superior region of the cervix progress into adenocarcinoma [283]. The primary risk factor associated with cervical cancer in women is HPV infection, which plays a crucial role in the development and progression of the carcinoma (Fig. 5B) [284]. The infection produced by this sexually transmitted oncogenic virus accounts for more than 99 % of the cases. Cervical cancer poses a significant health concern for women due to the significant time lag between infection and malignancy.

Cervical cancer undergoes a sequential progression, mostly determined by the morphological characteristics of cervical dysplasia in lesions. However, it is important to note that these lesions can remain and advance, while a considerable proportion may regress and resolve [285]. At present, the identification of cervical disease by pathological features is limited in its ability to definitively determine the underlying risk for the advancement of the observed cervical abnormality or lesion. However, clinical treatment regimens utilise risk models that rely on cervical pathology. On the other hand, HPV testing is appropriate for determining the specific type of HPV that is present, but it is unable to identify the cervical lesions. Therefore, it is currently necessary to employ an additional method that identifies cellular and/or molecular abnormalities that can predict the development of cervical cancer. Techniques like cytology and histology can achieve this. This additional approach is crucial in distinguishing women who are at a high risk of developing cervical cancer and require lesion ablation [286].

Fortunately, there is evidence to suggest that the timely identification and efficient management of cervical cancer have a substantial impact on reducing mortality rates among women. In industrialised nations, there has been a notable decrease in mortality rates associated with cervical cancer over the past four decades as a result of successful screening and immunisation initiatives [287]. Existing screening techniques for early detection of this condition include the PAP smear test, visual inspection with acetic acid (VIA), liquid-based cytology (LBC), and HPV testing specifically designed for high-risk HPV strains [288]. In the context of cervical cancer treatment, chemotherapy, radiation, and surgery are frequently employed [288]. However, it is important to note that these treatment modalities do not significantly enhance patient survival rates. Furthermore, significant advancements have been made in the domain of HPV vaccine development, resulting in a notable decrease in the likelihood of acquiring HPV infection [282]. Despite significant progress in the detection and prevention of cervical cancer, it remains a global health issue, as described by Small et al. [283], especially in undeveloped and emerging nations. This scenario is influenced by various variables, including limited awareness, socioeconomic circumstances, and the absence of affordable and easily accessible screening and immunisation initiatives [286]. The aforementioned variables have rendered the existing screening methods unfeasible, hence necessitating the development of more streamlined and easily available alternatives.

Due to the non-human source of HPV-DNA, it is possible to employ tiny panels or single-site assays in LB methodologies, which offers significant benefits in terms of cost-effectiveness and sensitivity. Hence, a majority of the studies conducted on individuals with cervical cancer have employed various PCR-based techniques, such as quantitative PCR or ddPCR approaches, which have demonstrated a sensitivity of less than 0.01 % [289]. Collectively, the identification of viral DNA in plasma and, in some instances, saliva in viral-associated malignancies has demonstrated a notable level of specificity and even the possibility of early detection. Moreover, when it comes to therapeutic usefulness, the presence of HPV-ctDNA appears to be strongly linked to unfavourable outcomes in cervical neoplasias. The utilisation of LB enables convenient sequential examinations, allowing for the monitoring of HPV-ctDNA as a potential indicator of treatment efficacy or inefficacy as well as a potential marker for the presence of persistent residual disease.

Epigenetic modifications, including methylation and histone acetylation, are significant genetic regulatory systems that also exert a pivotal influence on the development of cancer [290]. Epigenetic modifications occur in both the host and HPV genomes during an HPV infection. Both E6 and E7 possess the capability to modify the DNA methylation patterns of infected cells. The E7 protein has the ability to directly interact with and initiate the activation of DNA methyltransferase 1 (DNMT1), leading to the methylation of several genomic host sites. DNMT1 is the primary enzyme responsible for preserving methylation patterns after DNA replication by facilitating methyl group transfer to cytosines. In addition, E6 has the ability to stimulate DNMT1 expression by suppressing p53. In addition, E7 has the ability to stimulate the presence of KDM6A or 6B, resulting in the demethylation of certain target genes, such as p16. People commonly use the overexpression of p16 as a proxy for human recombinant human papillomavirus (hrHPV) infection and transformation, along with the proliferation marker Ki67. Typically, when screening tissues and LBC for cervical cancer, a higher level of methylation in both the host and viral sequences has been linked to enhanced invasiveness [291,292].

As an early and specific epigenetic event in cervical carcinogenesis, methylation serves as a crucial clinical tool for early illness detection and diagnosis. Nevertheless, the examination of methylation status in LB samples obtained from cancer patients remains a complex task, requiring further methodological enhancements [293].

When the HPV genome is added to cervical cells, it often causes the transcripts that code for the viral oncogenes E6 and E7 to be upregulated and better preserved. This integration exhibits a preference for commonly vulnerable locations [294] and is recognised for its ability to cause DNA damage, anomalies in centrosomes, and mis-segregation of chromosomes, resulting in chromosomal instability. Consequently, it is not unexpected that cervical malignancies have a significant level of chromosomal changes and a pattern of APOBEC cytidine deaminase mutagenesis [295]. In the Cancer Genome Atlas, the comprehensive molecular analysis of all tumours revealed that cervical squamous tumours formed clusters with substantial aneuploidy. These clusters were characterised by significant changes in proliferation, DNA repair pathways, and basal signalling.

Typically, tumours exhibit distinct mutation patterns, with just a few genes being shared across all tumours. However, similar to other types of carcinomas, ERBB2/PI3K/AKT/mTOR are particularly impacted. Three comprehensive studies utilising whole-exome sequencing to analyse cervical tumours have been published [296]. Even though the target genes had very different mutation rates, all of the studies found that hotspot mutations were present in more than 10 % of the samples. These mutations were in the serine/threonine protein kinase PIK3CA gene.

Single-gene ctDNA techniques are typically not practical due to the significant variation in mutation patterns observed in cervical neoplasias. Nevertheless, by utilising deep sequencing methodologies that analyse extensive gene panels, it is possible to achieve notably elevated sensitivities, even in individuals diagnosed with cervical cancer. Moreover, these mutations serve as indicators of a more unfavourable illness outcome in terms of both disease progression and survival. Nevertheless, these methods are costly and necessitate adjustments for potential CH, thereby further increasing expenses. Hence, it is highly probable that a multi-analyte method is more sensitive in routine clinical approaches for cervical neoplasias.

Han et al. proposed in a recent study that a NGS methodology could surpass PCR-based technologies in detecting viral ctDNA [297]. Therefore, the scientists have devised an innovative NGS approach for the detection of viral ct-DNA in both HPV and Epstein-Barr virus (EBV). This method utilises a hybrid capture methodology to perform genome sequencing for both viruses. The study showed that the NGS approach for detecting HPV in CEC patients had a detection threshold that was 10 times lower than digital PCR. Additionally, it exhibited a sensitivity and specificity of 100 %. The study showed that viral genome NGS offers significantly higher sensitivity compared to PCR-based techniques for virus-associated malignancies. Additionally, it allows for viral subtyping and examination of ct-DNA fragment length.

Viral DNA fragments in blood have been demonstrated to be a highly specific and sensitive liquid biopsy tool in virus-associated cancer, namely Epstein-Barr virus-related nasopharyngeal carcinomas [298]. Epigenetic modifications can function as very sensitive liquid biopsy indicators, in addition to detecting circulating HPV DNA. These changes are not affected by the confounding effects of clonal hematopoiesis. Certain promoter methylations have been recognised as significant facilitators in the progression of high-grade dysplastic lesions (HSIL/CIN2-3) to invasive carcinomas in cervical cancer [299]. Researchers have extensively studied the promoter methylation of genes such as cell adhesion molecule 1 (CADM1) and myelin and lymphocyte protein (MAL) in cervical tissue and smears. This phenomenon has been commonly found in the progression of cervical cancer [300]. CADM1 is recognised as a gene that inhibits tumour growth and has a role in the contacts and attachment between cells [301]. The MAL gene is commonly recognised as a tumour suppressor gene in numerous types of cancer. The involvement of MAL in apical sorting and raft stabilisation has been suggested [302]. In the future, the utilisation of combined detection of methylation of both genes in cfDNA has the potential to serve as a readily accessible alternative molecular monitoring tool for women who are positive for hrHPV.

Fabinshy Thangarajah et al. [303] demonstrate the feasibility of detecting ccfHPV-DNA in cervical and vulvar cancer by using ddPCR. It has great potential for monitoring therapy in advanced stages of cancer, as it indicates the response to treatments, and the number of copies is associated with the amount of tumour present. Its effectiveness as a marker for therapy monitoring or a guide for MRD-guided therapeutic algorithms should be assessed in prospective clinical trials. Novel therapeutics targeting HPV are of great interest.

In the detection and monitoring of cervical cancer, serum tumour biomarkers such as CEA, SCC Mag, and CA19-9 have been commonly employed due to their non-invasive measurement in blood samples [304]. Nevertheless, none of these methods provide the necessary specificity to identify cervical cancer, nor do they exhibit the necessary sensitivity for early-stage cervical cancer diagnosis [304]. In recent times, there has been a growing endorsement of miRNAs as promising biomarkers for the non-invasive identification of cervical cancer, exhibiting notable sensitivity and specificity [305]. MiRNAs are a class of non-coding RNAs consisting of 20–24 nucleotides. The deregulation of miRNAs in cancer has been observed to occur often [306]. Recent research has identified eight miRNA biomarkers, specifically miRNA-20a, miRNA-205, miRNA-218, miRNA-21, miRNA-29a, miRNA-200a, miRNA-25, and miRNA-486-5p, as having the ability to differentiate between those diagnosed with cervical cancer and those who are considered healthy [106].

In their study, Causin et al. [307] successfully found a total of nine miRNAs that exhibited the ability to differentiate between the HFS and CIN-3 groups. These miRNAs include miR-205-5p, miR-130a-3p, miR-3136-5p, miR-128-2-5p, let-7f-5p, miR-202-3p, miR-323a-5p, miR-381-3p, and miR-4531. Moreover, the researchers demonstrated that the nine miRNAs under investigation do not exhibit any association with HR-HPV infection. This suggests that the differential expression of these miRNAs may be a contributing factor unrelated to HR-HPV infection. The researchers also used multiple logistic regression analysis to find four specific miRNAs (miR-205-5p, miR-130a-3p, miR-4531, and miR-381-3p) that might be useful as biomarkers for CIN 3 in samples of cerebrospinal fluid (LBC) made from white blood cells. Our in silico functional experiment results suggest that these miRNAs may contribute to the progression of CC.

Multiple investigations have found multiple nuclear transporters in the exosomes of CC cells, which have also been confirmed in serum. These transporters have been pooled as a collection of biomarkers and have been recognised as prospective diagnostic biomarkers. The serum exosomal lncRNA DLX6-AS1 levels in patients with CC were shown to be significantly elevated in comparison to patients with CIN and normal controls [308]. Patients with CC showed a significantly lower plasma exosomal miR-125a-5p expression level compared to healthy controls. This finding suggests that CC patients may have a potential marker for distinguishing between noncervical cancer and cervical cancer [303].

Exosomes facilitate the transportation of miRNAs, lncRNAs, and other functional RNAs between cells. Exosomal miR-1323 has been observed to be secreted by CAFs and subsequently transferred to CCs, thereby facilitating the progression of CC and enhancing radioresistance [309]. Conversely, exosomal miR-1468-5p, released by CC cells, has been found to enhance tumour immune evasion by exerting immunosuppressive effects through lymphatic endothelial cells (LECs) in the TME. It is worth noting that elevated levels of serum exosomal miR-1468-5p are associated with an immunosuppressive state and an unfavourable prognosis in patients with CC [310]. CC cells release exosomal miR-142-5p, which also suppresses the immune response by decreasing the production of indoleamine 2, 3-dioxygenase by LECs [311]. The promotion of EMT and metastasis in CC cells is facilitated by exosomal miR-663b, which specifically targets MGAT3 in response to TGF-β1 stimulation [312]. In exosomes, lncRNAs have been identified. Specifically, exosomal lncRNA UCA1, derived from cancer stem cells, has been observed to facilitate the self-renewal and differentiation of CC stem cells by activating the miRNA-122-5p/SOX2 axis [313]. Similarly, exosomes originating from cancer cells have been found to produce lncRNA AGAP2-AS1, which plays a regulatory role in the miR-3064-5p/SIRT1 axis, thereby promoting the proliferation of CC cells [314]. Additionally, exosomes have been found to transfer LncRNA LINC01305 to recipient cells, thereby enhancing the progression of CC [315].

Studies have also suggested the involvement of exosome-carrying proteins in the pathogenesis of CC. For instance, exosomes transport the Wnt2B protein from CC cells to fibroblasts. Once inside, it stimulates the activation of fibroblasts into CAFs and promotes the progression of CC [316]. The presence of HPV E6 transcripts in the exosomes of CC cells has been observed, suggesting that these transcripts could potentially function as exosome biomarkers for CC [317]. Based on these examples, it is evident that exosomes significantly contribute to the advancement of CC. Furthermore, it is worth investigating the clinical significance of exosomes in CC diagnosis and treatment.

### 2.3 Endometrial cancer (EC)

EC is a prevalent malignancy in affluent countries, with its occurrence on the rise over the past decade [318]. Globally, researchers identify a staggering 142,000 cases annually, with a mortality rate exceeding 2.3 per 100,000 women [319]. The prevalence of this gynaecological condition exhibits a positive correlation with advancing age, as seen by the average age of diagnosis being 61 ± 2 years. Furthermore, ninety percent of patients manifest after the age of 50 [320]. Despite the favourable prognosis observed in the majority of patients with the disease as a result of timely detection, approximately 15–20 % of these tumours demonstrate an aggressive character [321]. Alarmingly, the mortality rate for EC has been on the rise over the previous two decades, with a notable increase of 8 % in the last 13 years [320].

The classification of this tumour has historically been based on tumour histology, resulting in two distinct categories of endometrial tumours [322]. Type I, referred to as oestrogen-related endometrioid carcinomas (EEC), accounts for approximately 70 % of EC cases and is distinguished by the presence of low-grade tumours that exhibit a favourable prognosis. Non-endometrioid endometrial cancer (NEEC), commonly referred to as Type II tumours, lacks oestrogen regulation and typically manifests as serous and clear cell carcinomas [4]. These types of tumours typically exhibit a more unfavourable prognosis [322]. In recent times, researchers have developed a molecular categorization for malignant cancers. The classification of EC into four distinct groups is determined by TGCA, which takes into account the existence of somatic mutations, copy number changes, and microsatellite instability status [323]. Tumours with inactivating mutations in POLE exonuclease are classified as the first group, referred to as POLE ultramutated. The term "MSI hypermutated" refers to a group of cancers that are hypermutated or microsatellite unstable (MSI). On the other hand, "copy-number low" (CN low) refers to tumours with low copy-number aberrations, while "copy-number high" (CN high) refers to tumours with high copy-number changes [324]. The final category primarily consists of serous tumours, typically distinguished by p53 mutations and an unfavourable prognosis [325].

Age ≥40 years, obesity, diabetes, hypertension, oestrogen use, tamoxifen medication, and family history of malignant tumours are among the risk factors associated with EC. Numerous factors show a strong correlation with contemporary lifestyles in developed countries. Implementing a successful screening approach for women with elevated risk factors could aid in the timely identification and treatment of EC. In developed nations, it may be beneficial to implement a screening programme that utilises liquid-based cytology for specific high-risk patient categories. The diagnostic protocol includes a pelvic examination and transvaginal ultrasonography, followed by a histopathologic examination of an endometrial biopsy. To obtain the biopsy, it is preferable to perform a minimally invasive aspiration from the uterine cavity using a Cornier pipelle, also known as a uterine aspirate or pipelle biopsy. The diagnosis is established through the examination of anomalous cells in the uterine aspirate, which has a notable level of sensitivity in detecting EC [326]. Nevertheless, there have been reports of elevated failure rates, averaging 22 % for histologically inadequate specimens. Consequently, a more intrusive diagnostic procedure such as dilatation and curettage (D&C) or hysteroscopy becomes necessary, accompanied by the additional hazards of anaesthesia, infection, and perforation, as well as increased healthcare expenses. The molecular subtype assignment between hysterectomy specimens and diagnostic endometrial specimens acquired through office biopsy (such as pipelle) or dilatation and curettage show a strong agreement [327].

Primary treatment for EC typically involves performing a total hysterectomy and a bilateral salpingo-oophorectomy. Omentectomy and retroperitoneal lymph node dissection are occasionally employed in certain instances [319]. Radiotherapy is employed as an alternative treatment for pelvic lymph-node regions that carry the potential for microscopic malignant tumours [319], including vaginal brachytherapy. Chemotherapy is the predominant treatment for metastatic or recurrent EC [328], with the recommended initial therapy being the combination of carboplatin and paclitaxel. However, despite recent endeavours to incorporate additional agents of interest, such as metformin, temsirolimus, and bevacizumab, into the established therapeutic protocol, the efficacy of these agents as initial treatment for advanced tumours does not surpass 40 %. It is worth mentioning that the past response rates to second-line chemotherapy, typically involving the administration of paclitaxel, have been rather low, with a reported rate of less than 20 % [329]. Hormone medication and immunotherapy are distinct targeted medicines that are presently accessible for the management of advanced EC, alongside conventional chemotherapy regimens [330].

**Fig. 6:**
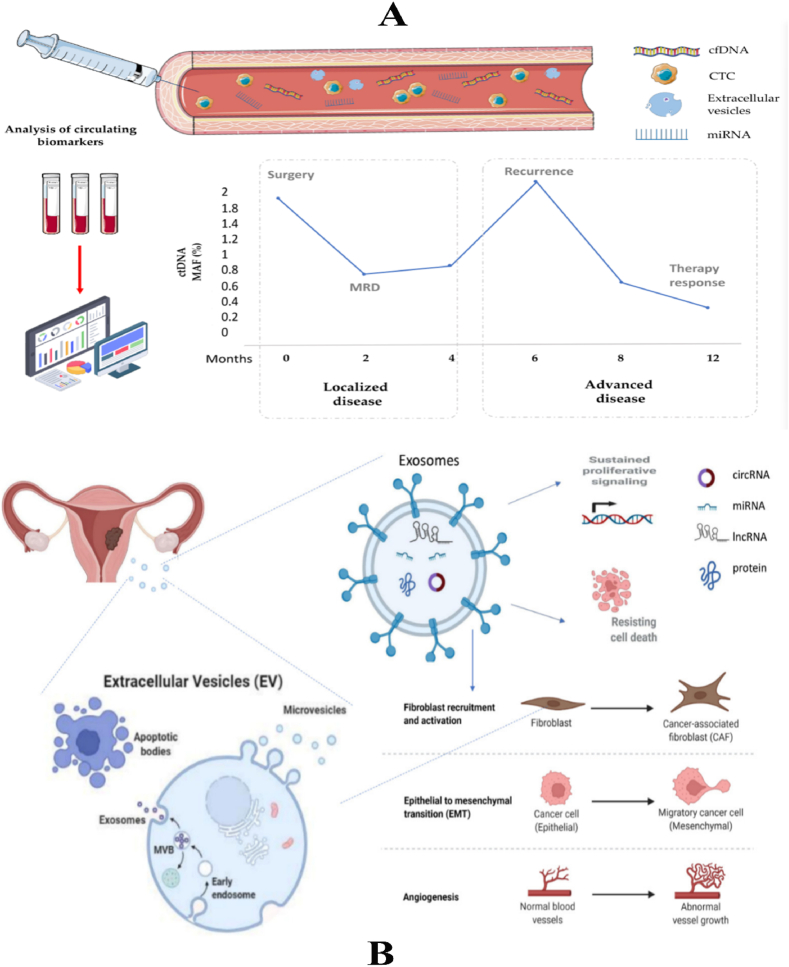
A. Clinical contexts for the application of liquid biopsy (CTCs, cfDNA, cEVs and miRNAs) to improve the management of patients with both localized and advanced EC [333]. B. The role of exosomes in the progression of endometrial cancer. Exosomes are released by EC cells as well as cancer-associated fibroblasts and tumor-associated macrophages. They contain molecules (proteins, miRNAs, circRNAs, and lncRNAs) that participate in cancer progression by enhancing tumor proliferation, inhibiting apoptosis, inducing angiogenesis and epithelial to mesenchymal transition [334].

Molecular investigations, like those conducted by the TCGA, have elucidated the topography of chromosomal changes present in EC. These studies have yielded vital insights into the aetiology of this disease [331]. Nevertheless, the current state of molecularly guided management for EC falls behind that of other prevalent malignancies. In the context of breast or lung cancers, a diverse range of molecular markers exist to inform the selection of treatment modalities. These possibilities encompass the utilisation of targeted medicines in conjunction with other therapeutic approaches for patients who have advanced or recurring disease [332]. Currently, there is a lack of established biomarkers used for the purpose of monitoring endometrial tumours. Given the increasing prevalence of the disease and the lack of effective methods to identify patients with the most unfavourable prognosis and responsiveness to treatment, it is imperative to find biomarkers that can predict recurrence and evaluate disease response. These indicators are crucial for implementing an individualised treatment approach that reduces the adverse effects linked to chemotherapy and radiotherapy while also optimising the identification of individuals who will benefit from targeted medications and immunotherapy (Fig. 6A).

#### 2.3.1 Exosomes in endometrial cancer

Exosomes' potential applications in endometrial liquid biopsy are significant. Exosomes derived from the peritoneal fluid, urine, and serum of patients with EC contain nucleic acids that could serve as novel diagnostic biomarkers for EC.

In comparison to other solid tumours, the characterization of exosomes in gynaecological malignancies, specifically EC, is rather limited [5]. There is currently a significant and continuous endeavour to investigate the involvement of exosomes in the development of EC. This is due to the substantial body of evidence that establishes a connection between exosomes and various aspects of EC, including angiogenesis and EMT, as well as the survival, growth, and invasive and metastatic capabilities of EC cells (Fig. 6B) [180]. EC cells, CAFs, and TAMs generate exosomes that play a crucial role in the communication between these cell groups, contributing to the pathophysiology of EC cancer.

The study utilised urine-derived exosomes obtained from individuals diagnosed with EC and patients exhibiting symptoms of EC but without confirmed EC to assess a distinct miRNA expression profile. Using qPCR, they successfully amplified 57 out of the 84 miRNA sequences examined, indicating the distinct arrangement of miRNA within exosomes. Furthermore, they determined that hsa-miR-200c-3p displayed the highest level of enrichment. They assessed the biological importance of hsa-miR-200c-3p in EC using a range of bioinformatics and in silico methods. Akhil Srivastava et al. assert that we can employ the identification of distinct miRNA patterns in exosomes to uncover biomarker signatures and diagnose EC. Among these candidates, hsa-miR-200c-3p stands out as a promising one. Exosomes produced from urine have the potential to facilitate the advancement of non-invasive biomarkers [335].

In patients with EC, there was a substantial increase in plasma-derived exosomal miR-15a-5p and exosomal lectin galactoside-binding soluble 3-binding protein (LGELS3BP) levels compared to the control group. Notably, the combination of miR-15a-5p with blood tumour markers (CEA and CA125) resulted in an AUC value of 0.899 [336]. Exosomal LGELS3BP also stimulates the development of EC cells and the angiogenesis of HUVEC [337]. Fan et al. looked for miRNA markers in the serum of people with EC and confirmed that they were the same in both the serum and the plasma exosomes. They identified exosomal miR-20b-5p and miR-151a-5p [336] as possible noninvasive biomarkers for diagnosing EC.

Proteomic analysis of plasma exosomes from individuals with EC revealed a high concentration of the lectin galactoside-binding soluble 3 binding protein (LGALS3BP). The development of ECs and the angiogenesis of human umbilical vein endothelial cells (HUVECs) are facilitated by LGALS3BP through the activation of the PI3K/AKT/VEGFA signalling pathway, both in vitro and in vivo. Patients with atypical endometrial hyperplasia as well as low-grade and high-grade EC had a gradual increase in LGALS3BP levels, which was associated with a negative prognosis. As a result, it is plausible that plasma exosome LGALS3BP could serve as a biomarker of diagnostic and prognostic significance [[66], [67], [68], [69], [70], [71], [72], [73], [74], [75], [76], [77], [78], [79], [80], [191]]. In addition, the Annexin 2 (ANXA2) protein is highly concentrated in plasma EVs obtained from patients with EC. Researchers have proposed ANXA2 as a potential diagnostic biomarker for EC due to its ability to accurately and sensitively differentiate between patients with EC. Additionally, there is a correlation between ANXA2 levels and non-endometrioid histological type, high-grade histology, advanced FIGO stage, and a heightened risk of recurrence. Hence, the detection of plasma EV ANXA2 is regarded as a potentially valuable biomarker with diagnostic and prognostic implications [168].

In contrast, it has been observed that EVs released by tumours play a significant role in facilitating intercellular communication between tumour cells and stromal cells. These EVs are involved in both local and distant microenvironments and were involved in the establishment of the pre-metastatic niche before CTC colonisation [338]. Tumour exosomes, which are a specific type of EV ranging in size from 40 to 150 nm, are released from tumour cells as a result of the fusion between multivesicular late endosomes or lysosomes and the plasma membrane. These exosomes have been demonstrated to accurately represent the tumour of origin [339]. Additionally, they play a crucial role in intercellular signalling processes, including EMT and the localization of disseminated disease to pre-conditioned tissues [340]. In this situation, EVs produced from ECs might serve as carriers for chemicals that are useful for early detection and monitoring of patients. Additionally, these EVs can actively contribute to the recruitment of tumour-supporting cells and CTCs in a microenvironment that promotes metastasis. Recent findings have indicated a rise in the release of EVs linked to severe illness [340]. The biological significance of tumour EVs in the advancement of diseases and their propensity for metastasis. Nevertheless, the task of determining the specific cell of origin and conducting a comprehensive biological analysis of circulating tumour EVs continues to pose significant difficulties.

Carolina Herrero et al.'s [168] study demonstrated that ExoGAG is a dependable, user-friendly, and highly effective technique for extracting EVs from a variety of biological fluids, such as conditioned culture media and plasma. Furthermore, the ExoGAG technique demonstrated exceptional performance in the clinical application of EVs derived from EC patients' plasma. The enhanced specificity and sensitivity of the separated EVs as a liquid biopsy were attributed to the targeted expression of the EC biomarker ANXA2. Furthermore, it is possible to detect elevated levels of ANXA2 in plasma EVs that are linked to non-endometrioid tumours and tumours with a high likelihood of recurrence. This finding suggests that expressing ANXA2 in EVs could serve as a valuable diagnostic and prognostic biomarker in EC.

#### 2.3.2 miRNAs in endometrial cancer

miRNAs are a class of post-transcriptional regulators that play a significant role in several cellular processes, such as carcinogenesis and treatment resistance. A solitary miRNA has the ability to selectively target various messenger RNA molecules and exhibit either oncogenic or tumour-suppressive properties. One notable characteristic of miRNAs is their notable stability in serum [137].

In recent years, there has been an increase in the investigation of miRNAs as potential biomarkers. MiRNAs have been found to be linked to the regulation of gene expression, epigenetic malfunction, and carcinogenesis in individuals diagnosed with EC. The potential of circulating free miRNAs as biomarkers for early EC diagnosis and tumour progression detection has been documented in previous studies [341]. Notably, multiple studies have detected miRNAs in EVs derived from various bodily fluids. Srivastava et al. assessed the potential of miRNAs derived from urine-derived exosomes as a diagnostic biomarker for EC. Their study revealed an enrichment of miR-200c-3p in urine exosomes from patients [342].

Patients with EC and individuals without the condition showed a notable increase in the expression of miR-15a-5p, miR-106b-5p, and miR-107 in their plasma exosomes, according to a comparative examination of the exosomal miRNA profile. Importantly, miR-15a-5p is a promising diagnostic biomarker for early EC that lets doctors tell the difference between people with stage I EC and those who don't have the condition. Furthermore, there is a correlation between the expression of miR-15a-5 and clinicopathologic characteristics such as tumour size, muscle layer infiltration, and positive p53 staining. However, there is no correlation between miR-15a-5 expression and a histological subtype that exhibits equal levels of expression in endometrioid or non-endometrioid histology in EC [336]. A different study with a similar goal found a group of microRNAs (miR-142-3p, miR-146a-5p, and miR-151a-5p) that are significantly more abundant in the plasma of people with EC than in people who don't have the condition. Researchers have proposed this 3-miRNA pattern as a potential diagnostic biomarker. Among the miRNAs examined, it was observed that only miR-151a-5p exhibited upregulation in the plasma exosomes of patients with EC [343]. Furthermore, researchers have found that the expression of miR-93 and miR-205 in serum exosomes has predictive significance in EC. The expression of miR-93 is notably increased in the blood of patients with EC, and this upregulation is associated with factors such as smoking, high tumour grade, advanced FIGO stage, metastatic dissemination, and shorter OS. On the other hand, the levels of miR-205 are notably reduced and exhibit an inverse correlation with smoking, lymph node metastases, advanced FIGO stage, and diminished OS.

The use of miRNA-loaded exosomes restores downregulated miRNAs in EC, suggesting a compelling avenue for targeted therapy. The examination of the EC miRNA profile gives insights into the specific targets of this methodology. Recent studies have demonstrated a significant reduction in miR-499a-5p in EC tissue and cell lines. Both in vitro and in vivo settings have observed the inhibition of EC proliferation by miR-499a-5p. This inhibition is achieved by targeting VAV3 and suppressing EC growth, angiogenesis, and metastasis [344]. In the context of EC, we could consider the potential augmentation of miRNA-499a-5p expression as a supplementary treatment strategy.

Jianing Yang et al.'s [345] primary goal was to develop a methodology for identifying EC-specific microRNA biomarkers in liquid biopsy samples, ultimately aiming to improve the timely detection of EC in women. They obtained endometrial fluid samples during patient-scheduled in-office visits or in the operating room prior to surgery, using the same procedure as saline infusion sonohysterography (SIS). The endometrial fluid specimens were subjected to the extraction of total RNA, which was subsequently quantified, reversed by transcription, and used in real-time PCR arrays. They conducted the research in two distinct stages: an initial exploratory phase and a subsequent validation phase. A total of 82 individuals were included in the study, from whom endometrial fluid samples were obtained and analysed. Phase I included 60 patients with non-cancer endometrial carcinoma, while phase II included 22 patients. They selected 14 microRNA biomarkers from the 84 miRNA candidates for further validation and statistical analysis in phase II, based on their highest degree of expression fluctuation during phase I. Three microRNAs, namely miR-429, miR-183-5p, and miR-146a-5p, exhibited a consistent and significant fold-change in upregulation. In addition, a total of four microRNAs (miR-378c, miR-475, miR-1321, and miR-362-3p) were identified with distinct characteristics. This study elucidated the feasibility of collecting, measuring, and identifying miRNA from endometrial fluid through a minimally invasive technique during a patient's in-person appointment. In order to evaluate the efficacy of these early detection indicators for endometrial cancer, it was imperative to conduct screening on a more extensive collection of clinical samples.

#### 2.3.3 LncRNAs in endometrial cancer

lncRNAs are believed to play a role in the development and advancement of EC tumours. In vitro studies have demonstrated that the exosomal long non-coding RNA Deleted Lymphocytic Leukaemia 1 (DLEU1) enhances the growth, movement, and invasive capacity of EC cells by controlling the miR-381-3p/E2F3 axis. When EC cells take in exosomal DLEU1, it lowers the activity of MiR-381-3p. This makes E2F3, which is miR-381-3p′s target gene, overexpress itself. Consequently, the suppression of DLEU1 and/or miR-381-3p and E2F3 downregulation interferes with this crucial pathway for the advancement of EC and could potentially serve as a therapeutic intervention [346].

#### 2.3.4 circRNAs in endometrial cancer

CircRNAs are a distinct category of non-coding RNAs. Initially considered unworthy of thorough investigation, they have recently garnered significant interest due to their association with carcinogenesis. Researchers have identified circRNAs as competitive inhibitors of miRNAs, disrupting their ability to attach to their targets. Furthermore, studies have demonstrated the high concentration of circRNAs in exosomes. A study that compared the exosomal circRNA profile of sera from people with EC and healthy controls found that 275 circRNAs had different levels of expression, with most of them being higher. We found that pathways related to neoplastic migration and invasion involved the majority of the circRNAs that exhibited differential expression. These pathways include the focal adhesion pathway, the ECM-receptor interaction pathway, and the control of the actin cytoskeleton pathway. The circRNAs hsa_circ_0109046 and hsa_circ_0002577 were identified as the most significant markers of differential expression and have the potential to serve as valuable diagnostic biomarkers [347].

#### 2.3.5 CTCs in endometrial cancer

CTC are released by every tumour. The utilisation of diverse methodologies for detecting CTCs, coupled with the limited number of participants in the studies, hinders a dependable comparison of the various investigations. According to previous studies [348], the utilisation of the EpCam-based approach revealed that a range of 7–60 % of patients exhibited the presence of CTCs at various stages of EC. Lemech et al. found that the CTC-positive group had a shorter time to relapse (20.3 months compared to 30.8 months), a higher incidence of non-endometrioid malignancies, and a greater frequency of tumours larger than 5 cm [349]. The study found a correlation between the presence of CTCs and deep myometrial invasion as well as lymph node involvement in early-stage endometrial cancer. Cervical involvement was found to have a substantial association with CTCs (83.3 % versus 11.8 %, p = 0.00), although no CTCs were observed during the initial cycle of treatment [348]. RTqPCR analysis revealed a gene expression profile in a high-risk endometrial cancer population, indicating the presence of CTCs with a plasticity phenotype, stemness, and EMT characteristics.

We have used the FDA-approved technology CellSearch to assess the presence of CTCs. The results consistently indicate that there is a small group of high-risk EC patients who have EpCAM-positive CTCs in their bloodstream when they are diagnosed. However, there have been only a few cohort studies conducted on this topic. One study, which included 28 patients with grade 3 EC and 7 % of them were CTC-positive, found a connection between positive CTCs and both deep myometrial infiltration and positive lymph nodes [350]. Similarly, a study indicated that 15 % (n = 40) of patients with high-risk EC tested positive for CTCs. These patients were reported to have cervical involvement [351]. Furthermore, there was no significant link between CTCs and blood CA125/HE4, and no CTCs were detected after the first cycle of conventional chemotherapy. The ENITEC Consortium conducted research with 32 high-risk EC patients who tested positive for CTCs, accounting for 22 % of the whole sample [352]. Another study reported that 60 % (n = 30) of patients with advanced EC tested positive for CTCs and had detectable tumour cells in their bloodstream. These patients were typically found to have non-endometrioid histology compared to endometrioid histology, a tumour size greater than 5 cm compared to less than 5 cm, an advanced stage of illness, and poorer survival rates [353]. While the identification of CTCs in the bloodstream could potentially aid in determining the likelihood of recurrence in patients with EC, evaluating the prognosis, and providing guidance for postoperative treatment, there is now no definitive information available. As a result, their usefulness in the clinical context is limited.

The feasibility of isolating CTCs from ovarian, endometrial, and cervical malignancies and growing them in vitro for a brief duration was established by Kolostova et al. through the utilisation of size-based enrichment, specifically the MetaCell method [354]. In a more recent study, peripheral blood samples from 92 patients who underwent a surgical operation were analysed using the same enrichment approach. The results showed a higher detection rate of CTCs compared to prior research. Furthermore, the authors asserted that the cultivation of endometrial CTCs was effectively accomplished, enabling subsequent functional and molecular characterization [355].

Sarah Francini et al. [356] demonstrated the possibility of detecting CTCs in blood samples taken from the ovarian vein of patients after laparoscopic surgery for EC. Ovarian vein blood samples from 80 % of patients exhibited the presence of CTCs; however, peripheral blood samples did not show any CTCs. Furthermore, ovarian vein samples revealed the presence of ER (+) CTCs and CTC clusters, which could have prognostic implications. There was no observed correlation between the CTC number and clinicopathologic features. Nevertheless, this study was conducted as a pilot, and it is necessary to confirm these initial findings in a larger group of individuals with early-stage EC, utilising larger quantities of peripheral blood (e.g., 20–30 mL). Finally, it is necessary to examine the predictive and potential therapeutic aspects of identifying CTCs during laparoscopy in patients with EC. One crucial aspect will involve evaluating the correlation between CTCs and/or the existence of ER (+) CTC/CTC clusters and the clinical outcome. This will be accomplished by utilising liquid biopsy samples obtained in proximity to the tumour.

#### 2.3.6 cfDNA/ctDNA in endometrial cancer

The process of collecting an collecting an endometrial sample has the potential to cause adverse effects such as pain, bleeding, infection, and uterine perforation. Additionally, it is worth noting that a biopsy alone may not provide a full diagnosis in approximately 25 % of instances [357]. The utilisation of ctDNA in liquid biopsy has been recognised as a significant biomarker for the timely detection and surveillance of cancer. It has been extensively investigated in several malignancies, including breast cancer, colorectal cancer, prostate cancer, lung cancer, and others [358]. The test can identify both genetic and epigenetic alterations in both plasma and urine samples. In this study, we conducted a comprehensive examination of the potential clinical utility of liquid biopsy in the diagnosis and treatment of EC.

The presence of cfDNA in the bloodstream is a consequence of cellular death. Over the past decade, there has been significant advancement in the isolation process of cfDNA, particularly with the implementation of NIPT (non-invasive prenatal testing). ctDNA is a distinct component of cfDNA that arises as a result of tumour apoptosis and necrosis. The sensitivity and specificity of ctDNA are enhanced due to its increased concentration in comparison to CTC, as well as the presence of somatic mutations in ctDNA that are distinct to a particular tumour [359]. An essential constraint of ctDNA is the requirement for a sufficient concentration of ctDNA to enable a dependable measurement. Furthermore, these techniques also depend on established mutations specific to each kind of cancer, which restricts their applicability as a comprehensive test for all types of cancer [360].

Researchers have linked changes in cfDNA levels to the onset and progression of cancer. However, research assessing the cfDNA concentration in individuals with EC is scarce. The feasibility of detecting cfDNA using the PCR-RFLP method and enriching it using the PCR-RFPL method was established by Dobrzycka et al. in a cohort of 109 patients diagnosed with EC, consisting of 87 patients with type I and 22 patients with type II [361]. TP53 mutations were detected in plasma within this cohort, particularly in early serous carcinomas, while grade 2 endometrioid tumours had a significant prevalence of KRAS mutations. This study was among the initial investigations that specifically examined EC and proposed the significance of cfDNA monitoring as a prognostic indicator and for choosing personalised treatment plans [361]. Tanaka et al. assessed the cfDNA of 15 people without any health issues, nine people with benign gynecologic illnesses, and 53 people with EC. The researchers employed RTqPCR to examine Alu sequences in free DNA fragments as surrogate markers. Their findings revealed that the levels of cfDNA in EC were generally higher compared to healthy and benign conditions. However, there were no statistically significant differences in cfDNA levels between different stages or histological grades of EC, nor were there any significant changes observed before and after surgery [362]. A recent study observed elevated amounts of total cfDNA and cfmtDNA in the serum of individuals with EC compared to those with benign lesions. This was analysed using a SYBR Gold test and qPCR, respectively. Crucially, it was noted that the magnitude of this rise was notably greater in high-grade EC [363]. The cfDNA integrity index was also investigated by the same group as a convenient and noninvasive biomarker that could offer further insights for the diagnosis, prognosis, and therapy stratification of cancer patients.

Epigenetic markers have exhibited significant promise in the detection and classification of several types of cancer. Indeed, researchers have identified the methylation state of several genes as a precise method for cancer detection. As an illustration, SEPT9 has shown utility in the detection of colorectal cancer, whereas MGMT methylation was found to be indicative of brain tumours [364]. Margolin et al. reported a distinct hypermethylation observed at the ZNF154 CpG island in EC tissues when compared to a control group of normal tissues. The validation of these tissue data for blood tests was also conducted in silico [365]. While the results are encouraging because methylation indicators offer benefits over point mutations, there is still a requirement for a standardised methodology to incorporate these markers into clinical practice.

The study conducted by Clara Mayo-de-Las-Casas et al. [366] involved the analysis of KRAS and PIK3CA mutations in matched surgical biopsies, blood samples, and cytology-negative peritoneal lavages from a cohort of 50 patients diagnosed with EC. They performed NGS on surgical biopsies and used a highly sensitive quantitative PCR technique to examine cfDNA from plasma and peritoneal lavages for KRAS and PIK3CA hotspot mutations. NGS analysis of biopsies revealed mutations in KRAS, PIK3CA, or a combination of KRAS and PIK3CA in 66 % (33 out of 50) of patients with EC. Out of the total, 19 instances exhibited unique mutations. Quantitative PCR revealed KRAS and/or PIK3CA mutations in the lavages of 9/19 (47.4 %) hotspot EC patients. Conversely, a mere 2 out of 19 blood samples (10.5 %) obtained from patients with hotspot EC yielded positive results. The mutations detected in cfDNA specimens consistently corresponded to those observed in paired biopsies. In a span of less than six months, one of the two patients who tested positive in both plasma and lavage succumbed. Ultimately, it is possible to do mutational analysis on peritoneal lavages and blood samples obtained from early-stage EC.

#### 2.3.7 Proteins in endometrial cancer

The risk factors for endometrial cancer have been investigated in relation to circulating protein biomarkers associated with reproductive functioning, insulin resistance, inflammation, and obesity [367]. Audet-Delage et al. conducted the initial identification of a correlation between pre- and post-operative metabolites of estradiol and the survival of endometrial cancer. An adverse connection was established between the levels of estradiol before and after surgery and the overall survival rate. Furthermore, it has been established that CA125 and HE4 possess prognostic significance in the context of endometrial cancer [360]. The Netherlands conducted a prospective trial that linked increased levels of CA125 to an advanced FIGO stage, profound myometrial invasion, and lymph node metastasis. Furthermore, the present investigation found a significant association between CA125 and unfavourable disease-related survival in both low-grade and high-grade endometrial cancer, as determined using a multivariable analysis (hazard ratio [HR] 3.62, 95 % confidence interval [CI] 2.15–6.09, p < 0.001) [368]. Retrospective studies have found a correlation between pre- and post-operative CA125 levels and a poor prognosis in uterine carcinosarcoma, which is now classified as poorly differentiated serous endometrial cancer [369]. Even after accounting for the patient's glomerular filtration rate (GFR), a multivariable study found HE4 to be a significant predictor of deep myometrial invasion (p = 0.0005) in endometrial cancer. The specificity and sensitivity of biomarker-based models for deep myometrial invasion were enhanced by the correction of HE4.

## 3 Prospects and challenges of liquid biopsy in gynaecological oncology applications

In comparison to standard tissue biopsy, liquid biopsy offers a more thorough means of capturing the variability seen in gynaecological oncology. The non-invasive nature and practicality of liquid biopsy enable the collection of several samples and the continuous monitoring of tumour progression over time, facilitating the detection of treatment resistance and informing the choice of individualised therapy.

The development of liquid biopsy has garnered significant attention over the past decade. The emergence of a highly sensitive, precise, and non-invasive methodology to address significant clinical inquiries pertaining to early detection, prognosis, treatment efficacy, and disease surveillance has generated considerable optimism, particularly for cancers exhibiting substantial heterogeneity, such as gynaecological oncology. This article provides a comprehensive review of recent studies pertaining to the use of liquid biopsy in the field of gynaecological oncology. Additionally, it emphasises the unresolved inquiries that persist and require further investigation. Although certain studies suggest that methods involving the measurement and characterization of CTCs, cfmiRNAs, and EVs offer valuable insights into the biological characteristics of gynaecological oncology, the published findings lack sufficient consistency to establish definitive conclusions regarding their practical clinical implementation.

### 3.1 Potential prospects and challenges in the application of CTCs analysis

Despite the growing evidence about the therapeutic effectiveness of liquid biopsy in gynaecological oncology, the use of CTCs in clinical settings continues to be a persistent problem due to various restrictions. Firstly, earlier detection algorithms had limitations in enumerating CTCs due to their scarcity. Researchers are making significant efforts to create reliable approaches for detecting CTCs, which aim to achieve both high separation efficiency and detection sensitivity. Not only that, but they are also studying how CTCs grow in various culture models, including CTC lines, CDXs, and organoids derived from CTCs [34]. Furthermore, due to the diverse nature of CTCs, existing methods for identifying specific subgroups of CTCs are still restricted to studying those that primarily contribute to metastasis in gynaecological oncology. New developments in single-cell analysis methods for CTCs could help classify CTCs better and make it easier to study what characteristics metastasis-competent CTCs have. Furthermore, the half-life of CTCs following venipuncture is roughly 4 h, a relatively short duration [370]. Hence, the implementation of CTC analysis is restricted to normal practice, particularly in institutions without specialised CTC analysis equipment. Even with these problems, CTC analysis could provide important information from both quantitative and qualitative points of view, which would speed up the progress of CTC research in the coming era of precision medicine.

### 3.2 Potential prospects and challenges in the application of EVs analysis

EVs have a big effect on how cells talk to each other because they can change the function of target cells by changing their surface proteins or carrying chemicals across the boundaries of cells. This role is prevalent across various types of tumours. EV activity plays a crucial role in various pathophysiological processes, including inflammatory responses, immunoregulation, carcinogenesis, tumour invasion, and metastasis, as mentioned earlier. Tumor-derived EVs have become a novel reservoir of circulating cancer biomarkers because of their ubiquitous presence in all bodily fluids and distinct molecular contents compared to non-tumor EVs. It is worth noting that cEVs exhibit larger quantities in body fluids when compared to other circulating elements like ctDNA or CTCs. Moreover, cEVs play a crucial role in safeguarding and stabilising their molecular cargo. Hence, cEVs exhibit significant potential as biomarkers for many tumour types, such as endometrial cancer and ovarian cancer. However, the widespread adoption of EV-based biomarkers in clinical settings is very far from being a reality.

One of the primary obstacles to enhancing the utilisation of EVs for cancer treatment, specifically in the context of gynaecological tumours, pertains to the constrained efficacy of the techniques employed for the extraction and characterization of EVs. Technical standardisation is absent, and there is a lack of evidence regarding the high specificity and sensitivity for routine clinical use. Standard techniques are necessary for several aspects, such as sample collection and processing, EV isolation, and the analysis of the EV molecular payload. This is crucial because it hinders the comparability of data gained from different research projects. The isolation methodologies for EVs are typically categorised into five distinct techniques: ultracentrifugation, polymer-based precipitation, immune-selection, density-gradient separation, and microfluidic. Many bodily fluids have the potential to integrate and implement these solutions. Nevertheless, each of these approaches possesses some constraints. People widely employ ultracentrifugation as the predominant technique for the isolation of EVs. Nevertheless, this method is characterised by its time-intensive nature, substantial sample quantities, and limited EV recovery. An optimal technique for isolating EVs in a clinical setting should facilitate straightforward use without the requirement of intricate apparatus and should be rapid and compatible with a wide range of samples. At present, there are readily available methods for the isolation of EVs from liquid biopsies that have demonstrated successful application in the context of gynaecological tumours. These technologies include ExoQuick, ExoSpin, and ExoGAG [3,47,84]. The findings derived from these technologies exhibit promise, yet the majority of investigations pertaining to endometrial and ovarian tumours have been conducted on restricted patient cohorts. Hence, it is vital to conduct extensive clinical trials employing advanced technologies in order to address pertinent inquiries regarding gynaecological tumours. The primary clinical requirement for OC is the verification of biomarkers for early detection and screening, as well as indicators that have prognostic and therapeutic significance. Clinicians in the field of EC necessitate novel and precise biomarkers to categorise individuals with an elevated likelihood of recurrence following surgery, as well as new indicators to direct the choice of therapy in metastatic scenarios. In the near future, it will be crucial to enhance cEV isolation technologies, employ multi-omics strategies to analyse their molecular cargo, and carefully choose larger and well-defined patient cohorts for studies. This will facilitate the clinical application of circulating EVs in the treatment of gynecologic tumours.

Another complex area of research involves the validation of EVs for the purpose of medicine delivery [371]. The potential for this approach is substantiated by the presence of tissue tropism in EVs, which is facilitated by surface chemicals that might potentially be utilised for precise tumour targeting and subsequent administration of drugs. Liposomes are a prime example of flexible drug delivery vehicles that are readily available in clinical settings [372]. We can adjust the loading of hydrophobic and hydrophilic medicines in both the lipid membrane and the inner space, respectively. Similarly, the functionalization of EVs presents a viable option for the advancement of diagnostic tests that can anticipate metastasis in specific organs, as well as for the creation of more effective medicines that can hinder metastasis. Exosomal integrins have demonstrated the ability to guide the colonisation of specific organs by combining with target cells in a manner that is distinct to each tissue [340]. The translation of these technologies into clinical practice for various tumour types, including gynecologic tumours, faces significant challenges such as the efficient functionalization of EVs, control over the yield and stability of the therapeutic cargo, methods for purification and production scaling, sustained delivery over extended periods that align with clinical timings, and the development of appropriate and specific preclinical study designs.

In order to successfully implement EV-based analysis in clinical practice, a significant scientific endeavour will be necessary, including both researchers in the field of basic science and physicians. Furthermore, it is imperative that this study adhere to the guidelines set forth by the International Society for Extracellular Vesicles (ISEV) and other relevant working groups that advocate for the standardisation of protocols. This standardisation aims to enhance the consistency of procedures and kits used in EV analysis [373]. Additionally, it is crucial to investigate pertinent clinical inquiries within sufficient clinical cohorts.

### 3.3 Potential prospects and challenges in the application of ctDNA analysis

At present, the development of an early tumour diagnostic tool based on ctDNA is hindered by various possible barriers. Early detection of tumours is crucial for effective therapy and minimising morbidity. Hence, the determination of cancer stage in the absence of prior knowledge regarding cancer-specific mutations poses a significant challenge and may not yield optimal results in evaluating all genes related to cancer. As technology continues to advance, we expect the cost of ctDNA analysis methods, including NGS, to decrease. Nevertheless, despite the decreased expense, the existing techniques may restrict the identification to particular genes or segments of genes rather than facilitating the assessment of all identified genes linked to cancer. Another problem with early detection is that ctDNA isn't shed enough in the early stages of disease or micrometastasis, which is mostly because the disease isn't as strong at that point. Hence, in order to advance the clinical utilisation of ctDNA analysis, it may be necessary to develop innovative methods for collecting samples in the future. These methods should aim to stabilise blood cells and minimise the presence of background DNA in serum or plasma samples. Clonal hematopoiesis, a potential obstacle, has not been observed in the diseased tissue or organ in an occult disease due to intricate ctDNA mutations [374]. Ultimately, the identification of several mutations has the potential to provide significant insights into the aetiology of cancer in an individual patient. However, this endeavour is hindered by the limited occurrence and specificity of ctDNA. Simultaneously, the mutant ctDNA may exhibit substantial false-positive results as a result of mutations that are not acquired from cancer.

Various obstacles impede the progress of ctDNA analysis, but specific inherent characteristics of ctDNA may enhance its potential as a diagnostic instrument. Plasma cfDNA derived from a neoplasm is classified as ctDNA, and its concentrations are elevated in cancer patients in comparison to those in individuals without cancer [201]. The majority of research has primarily concentrated on genomic modifications for tumour surveillance, neglecting alterations in ctDNA fragment lengths. So, looking at fragment size, CNV-based screening, mathematical algorithm models, and ctDNA-associated methylation can help find ctDNA and might provide an alternative way to predict how cancer will progress.

### 3.4 Potential prospects and challenges in the application of DNA methylation analysis

DNA methylation is a significant epigenetic modification that holds a crucial position in the regulation of gene expression and cellular function. It is influenced by a complex interplay of genetic and non-genetic variables [375]. Recent DNA methylation studies in BC, OC, and CC have presented important evidence for understanding carcinogenesis. These findings offer prospective biomarkers for diagnosis and targets for therapeutic interventions. Nevertheless, DNA methylation has not yet attained the status of being a reliable indicator in specific tumours. This is primarily attributed to the nascent stage of the technology and the limited comprehension of the interactions between tumours and methylation. DNA methylation is an important component of genomic and epigenomic research. By integrating DNA methylation with other epigenetic alterations, such as histone modifications, it is possible to gain valuable insights. Studying the interplay between DNA methylation and other epigenetic alterations, such as histone modifications, can enhance our comprehension of the mechanisms that control gene expression and cellular function [376]. The role of DNA methylation in illness development is significant, and examining alterations in individual DNA methylation patterns can offer tailored diagnostic and therapeutic interventions for people. Subsequent investigations into DNA methylation will further enhance our comprehension of gene expression and biological mechanisms and are anticipated to yield novel advancements in disease therapy and personalised medicine. The fundamental objective of DNA methylation biomarker investigations in BC cases is to ascertain the identification of cancers exhibiting a negative prognosis and distinguish them from cancers with a favourable prognosis, as well as benign tumours. On the other hand, clinical testing for OC is necessary to identify all pathological types. The primary objective of clinical testing for CC is to identify the condition at its earliest stage.

The utilisation of highly specific biomarkers, together with other genetic markers derived from somatic and germ cells, holds promise for the management of personalised diseases, the monitoring of treatment responses, and the exact adjustment of dose. Consequently, this presents an opportunity to enhance treatment strategies and mitigate the occurrence of adverse effects. Furthermore, it has been demonstrated that epigenetic therapies possess the capacity to effectively address haematological and solid malignancies. When employed in conjunction with immunotherapy, these medicines have the potential to significantly enhance the efficacy of cancer treatment. By utilising methylation gene chips and bisulfite sequencing, we can identify the appropriate molecular markers. Methylation detection technologies serve as a means to facilitate clinical translation, enabling the early diagnosis of female cancer alterations.

WGBS (Whole-Genome Bisulfite Sequencing) is now considered the most reliable method for second-generation sequencing. However, due to its expensive nature, it may not be feasible for all researchers. Reduced representation busulfite sequencing (RRBS) is now considered to be the most reliable method. However, because of the expensive nature of WGBS, it may not be feasible for all researchers. Several triple sequencing approaches have evolved in recent years, including SMRT (single molecule real-time) and nanopore sequencing. These technologies show great promise in triple sequencing. Third-generation sequencing platforms based on nanopores have significant potential for direct sequencing at the 5 mC single-molecule level. Currently, the focus of DNA methylation development has shifted from generating large amounts of data to analysing it. Bioinformatics plays a crucial role in the development of epigenomic databases.

Implementing minimally invasive follow-up measures for gynecologic malignancies is crucial for promoting personalised treatment in this field. In this review, we have provided a summary of the scientific evidence regarding the possibility of various circulating biomarkers enhancing surveillance and disease monitoring for both non-advanced and advanced cancers. Furthermore, it is imperative to establish resilient preclinical models that can accurately replicate the molecular properties of tumours while considering the diverse nature of gynaecological oncology. This is crucial in order to authenticate novel targeted therapeutics in a personalised manner. Various pre-clinical models have been devised for the purpose of drug screening in the field of gynaecological oncology. These models encompass a spectrum, spanning from traditional two-dimensional cultures to patient-derived xenografts (PDXs). Nevertheless, 2D models lack sufficient representation of the diverse characteristics inside a tumour and are unable to accurately replicate the particular interactions between the tumour and stroma [[79], [80], [81], [82], [83], [84], [85], [86], [87], [88], [89], [90]]. Patient-derived organoids (PDO) possess the ability to maintain a portion of the tumour structure and molecular diversity, resulting in more precise patient-specific reactions compared to basic cell line models. Additionally, PDO eliminates the need for costly and time-consuming mouse models. Significantly, there has been a new generation of PDOs in the field of gynaecological oncology, which have been utilised for the evaluation of cytotoxic drugs. This has showcased their potential as a vital tool in preclinical research. PDO models play a crucial role in enhancing the understanding of tumour microenvironment interactions that influence the response to therapy, particularly in the context of immunotherapy. As an illustration, Bi et al. have successfully constructed PDOs using ovarian and endometrial tumours, thereby showcasing the predictive capabilities of these models in relation to medication response. It is worth mentioning that the examination of longitudinal liquid biopsies holds significance in informing the process of therapy selection screening in gynaecological oncology PDOs based on real-time genomic data. Additionally, it serves as a valuable resource for obtaining cellular material to generate organoids and accurately represent the molecular diversity of tumours.

Metabolomics, the study of metabolism at the global, or omics, level, has the potential to contribute considerably to biomedical research and, ultimately, to clinical medical practice. Metabolomics using MS allows for the simultaneous evaluation of various metabolite levels. This technique has proven to be highly influential in studying biomarkers, diagnosing diseases, measuring treatment responses, and identifying disrupted pathways caused by disease or treatment. MS-based quantitative analysis and biomarker identification utilising the metabolomics approach represent one of the primary platforms in clinical disciplines, including prognosis or diagnosis, assessment of severity, and response to therapy in a number of clinical disorders.

To summarise, liquid biopsy has become a prospective substitute for conventional tissue sample techniques due to its potential effectiveness in detecting and treating gynaecological oncology at an earlier stage. However, current evidence suggests that using liquid biopsy as a secondary or supplementary diagnostic modality could be more effective than using it as the sole biomarker to determine clinical intervention. Smaller sample sizes currently constrain most research on liquid biopsy techniques. In order to provide more robust evidence before implementing these techniques in routine clinical settings, it is imperative to conduct larger and higher-quality studies.

## 4 Conclusion

Liquid biopsy is on the verge of becoming a reliable and less-invasive method for diagnosing and managing gynaecological oncology. In addition to their diagnostic utility in cases where tumours are difficult to access, blood-based exosome assays have the potential to enable real-time monitoring of tumour progression and assessment of treatment effectiveness. While the majority of biomarkers now in use await prospective validation, the emergence of non-invasive liquid biopsy has already facilitated advancements in early identification, therapeutic response evaluation, prognosis, and patient outcomes in the field of gynaecological oncology.

Liquid biopsy has emerged as a viable option in the clinical context for some cancer types, including breast, colorectal, and prostate cancer. However, it continues to have significant potential in the field of gynaecological oncology. Large clinical trials are necessary to validate the analytical and clinical value of liquid biopsy-related technologies in endometrial cancer, such as specific cohort designs (e.g., multicenter or cohort size), the cohort dataset is connected to liquid and tissue biosamples biobanks allowing to unfold the translational potential of the multi-center pan-cancer cohort. These technologies include diagnostic and screening tools that rely on tumour material in uterine aspirates, as well as prognostic and monitoring tools that rely on tumour material in circulation, such as CTCs or ctDNA. In addition, the potential integration of various and complementary liquid biopsy techniques for surgical stratification and follow-up in patients with intermediate/high-risk and advanced EC could lead to a comprehensive approach that focuses on identifying mutations to assess residual disease, detecting recurrence at an early stage, selecting personalised therapies, and addressing disease relapse caused by therapy resistance. The use of well-designed and validated procedures in clinical trials, which encompass precise technical and cost-effective evaluations, is a promising avenue for the application of precision medicine in the field of gynaecological oncology.

The concept of the "liquid biopsy" holds significant promise in enhancing our understanding of cancer, encompassing the assessment of temporal and spatial heterogeneity. These factors pose considerable obstacles to the advancement of personalised anticancer treatments. Therefore, the use of liquid biopsy holds the potential to evaluate tumour heterogeneity more frequently and with decreased morbidity in comparison to tissue biopsies. Consequently, their advancement holds the potential to enhance the detection and surveillance of tumours, as well as the identification of precise treatments guided by precision medicine. Enhancing these specific concerns is a crucial determinant in mitigating the morbidity and mortality associated with gynaecological oncology. Hence, we will delve deeper into the latest advancements in precision oncology within the realm of gynaecological oncology. Specifically, we will explore how the enhancement of understanding in "liquid biopsy" research is facilitating the progress of targeted anti-cancer treatment and disease surveillance.

The process of developing a cancer biomarker and integrating it into clinical practice necessitates a multi-stage method and represents the culmination of extensive and labour-intensive research endeavours. Nevertheless, it is imperative to address several preanalytical, analytical, and post-analytical challenges while also doing research on assay validations pertaining to repeatability and reproducibility. Despite the extensive research conducted in the field of gynaecological oncology over the past few decades, the persistently low survival rates among patients can be attributed to the absence of reliable biomarkers for early identification, prognosis of clinical outcome, and responsiveness to treatment. Liquid biopsy methods are characterised by their minimal invasiveness and the ability to obtain serial sample readings that can be comfortably tolerated during the duration of treatment. This has the potential to contribute to the development of more effective personalised therapeutic algorithms and the monitoring of therapy in real-time. However, it is important to consider some obstacles that are unique to liquid biopsy studies. These challenges include pre-analytical considerations such as sample volume, appropriate tubes for sample collection, sample storage, analysis timing, quality control, and analytical validation of the assays.

Liquid biopsy has demonstrated its clinical importance in various cancer types, including gynaecological oncology. Nevertheless, the separation and detection of gynaecological oncology patients in the bloodstream lack standardised methodologies, and only a limited number of studies have successfully recruited large cohorts of patients. Prior to implementing liquid biopsy techniques in clinical routine, it is crucial to conduct additional research on the validation, standardisation, and quality control of the assays employed.

## CRediT authorship contribution statement

**Yingfeng Zhang:** Writing – review & editing, Writing – original draft. **Libi Tian:** Supervision.

## Informed consent statement

Not applicable.

## Data availability statement

All data analysed during the current study are available from the corresponding author upon reasonable request.

## Funding

This work was funded by project of Science and Technology Research Project of 10.13039/501100007957Chongqing Education Commission, KJQN202200462, study on the effect and mechanism of LncRNA RMST and Notch signaling pathway on trophoblast cells. This work was also funded by project of 10.13039/501100005230Natural Science Foundation of Chongqing Municipality, cstc2021ycjh-bgzxm0014, Study on the Role and Mechanism of Notch Signalling Channel in Regenerative Repair of Endometrium by Human Amniotic Mesenchymal Stem Cells.

## Declaration of competing interest

The authors declare that they have no known competing financial interests or personal relationships that could have appeared to influence the work reported in this paper.
